# Blocking calcium-MYC regulatory axis inhibits early dedifferentiation of chondrocytes and contributes to cartilage regeneration

**DOI:** 10.1186/s13287-025-04483-3

**Published:** 2025-07-15

**Authors:** Xu Wu, Yaoyao Fu, Jing Ma, Honglei Wang, Chenlong Li, Yaying Zhu, Qixuan Wang, Xudong Guo, Tianyu Zhang, Aijuan He

**Affiliations:** 1 Department of Facial Plastic and Reconstructive Surgery, Eye & ENT Hospital, Fudan University, Shanghai, 200031 China; 2 ENT Institute, Eye and ENT Hospital, Fudan University, Shanghai, China; 3 NHC Key Laboratory of Hearing Medicine, Fudan University, Shanghai, 200031 China; 4 National Stem Cell Translational Resource Center, School of Life Sciences and Technology, Tongji University, Shanghai, 200092 China

**Keywords:** Chondrocyte, Dedifferentiation, Single cell sequencing, Calcium signaling pathway, MYC

## Abstract

Tissue engineering technology for cartilage regeneration has increasingly emerged as a preferred method for repairing cartilage defects. However, the loss of chondrocyte-specific phenotypes during in vitro expansion, commonly referred to as dedifferentiation, impedes cartilage regeneration. Current research has yet to fully elucidate this phenomenon, hindering the development of improved cartilage regeneration. Our study employed single-cell sequencing and transposase-accessible chromatin sequencing to identify biomarkers, cell lineages and cellular characteristics within auricular chondrocytes during in vitro expansion. Our results showed that lower passage (P3) chondrocytes exhibited more dedifferentiated phenotypes with increased chromatin accessibility, while higher passage (P6) chondrocytes demonstrated hypertrophic characteristics. Furthermore, we identified that increased calcium influx was closely associated with the early dedifferentiation of chondrocytes, while inhibiting calcium signaling in early dedifferentiated cell could reverse cell phenotypes and promoted cartilage regeneration. In-depth mechanism research revealed that the expression of *MYC* mRNA was downregulated by increased calcium influx, which subsequently reduced *SOX5/SOX6* levels, important transcription factors for chondrocytes, leading to diminished extracellular matrix production and early dedifferentiation. In conclusion, we provide a comprehensive understanding of chondrocyte dedifferentiation and propose new strategies for optimizing cartilage regeneration systems.

**Graphical abstract:**

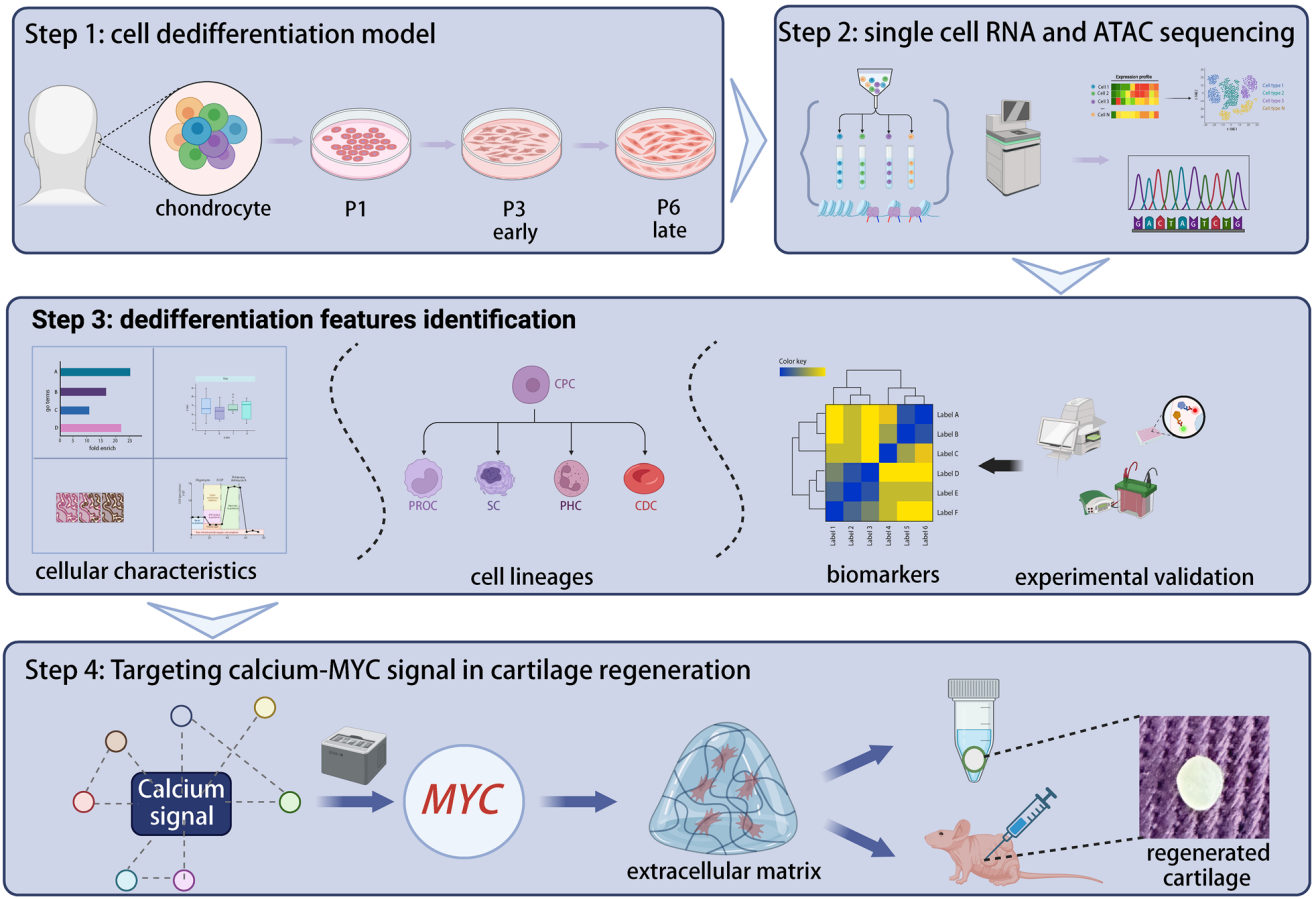

**Supplementary Information:**

The online version contains supplementary material available at 10.1186/s13287-025-04483-3.

## Introduction

Cartilage defects, such as congenital microtia [1] and joint [2] defects, are common clinical occurrences. Currently, the most prevalent methods for cartilage repair include ACI (autologous chondrocyte implantation) [3, 4] and synthetic material substitutes [5]. However, these methods are severely limited by issues such as donor site morbidity, lack of functionality, and immune rejection. Tissue engineering technology, which constructs functional tissues from a small amount of autologous cells to achieve biologically active autologous tissue or organ regeneration, has emerged as a focal point in regenerative research [6]. Despite this, the clinical translation of engineered cartilage has not yet achieved a breakthrough [7, 8], primarily due to the loss of chondrocyte phenotypes during in vitro culture [9, 10].

Historically, autologous chondrocytes that are extensively expanded exhibit a loss of phenotypes characterized by decreased type II collagen expression and extracellular matrix secretion [11, 12]. Consequently, regenerated cartilage tissues are suboptimal and have even exhibited characteristics of ossification and fibrosis in long-term clinical trials [7, 12, 13]. This phenomenon, referred to as "dedifferentiation," has been recognized since 1972 [14]. Dedifferentiation typically refers to the process by which mature cells lose their specialized characteristics and revert to a more primitive state [15, 16]. However, research findings have not been entirely consistent, as decreased proliferative capacity [17], characteristics similar to mesenchymal stem cells, and enhanced osteogenic potential have all been observed [18]. At higher passages, cells may even exhibit features of senescence [9]. These findings suggest an urgent need for more comprehensive data to precisely investigate this process.

Several studies indicate that factors such as methylation levels [19] or mechanical stress [20] may influence chondrocyte phenotypes in vitro. Single-cell sequencing of mouse chondrocytes has revealed the presence of functionally distinct subpopulations, which exhibit varying proportional changes with increasing passage number, indicating that aerobic metabolism may significantly influence this process [17]. Additionally, chondrocyte phenotypes can be reversed in lower passages but not in higher ones [17, 21, 22]. The limitations of previous studies, including the small number of cells analyzed via Smart-seq, leave significant questions unresolved regarding the specific underlying mechanisms. It remains unclear whether the single-cell sequencing findings in mouse chondrocytes are applicable to humans.

Overall, several critical issues need to be addressed: (1) High-resolution single-cell maps of human chondrocytes for interpreting cell lineages; (2) Precise biomarkers and specific cellular changes for human chondrocyte dedifferentiation which may explain why chondrocyte phenotypes can be reversed in lower passages but not in higher ones; (3) Specific signals regulating cell dedifferentiation and methods for reversing cell phenotypes.

Our team has been dedicated to ear reconstruction in children with congenital microtia for more than 20 years. Therefore, in order to promote the clinical transformation of cartilage tissue engineering technology in ear reconstruction, we chose auricular cartilage as our research object. To address these above-mentioned questions, our study selected primary (1st), lower passage (3rd), and higher passage (6th) cells for investigation, based on our team's previous dedifferentiation model [11, 23]. Utilizing 10 × genomics single-cell sequencing, we constructed a high-resolution map of human chondrocytes and identified biomarkers for dedifferentiation. Through differential analysis of the three passage groups and chromatin accessibility sequencing, we clarified that in vitro expansion of cells leads to a transition from dedifferentiated phenotypes in lower passages to hypertrophic phenotypes in higher passages. Moreover, by employing multiplex bioinformatics analysis and experimental validation alongside the cut&tag method, we discovered that increased calcium influx reduces MYC mRNA expression, subsequently diminishing the expression of downstream matrix-associated *SOX5/SOX6* genes. This mechanism is likely a primary factor driving early chondrocyte dedifferentiation.

## Results

### Cell lineages and differentiation trajectories from lower to higher passages

**Fig. 1:**
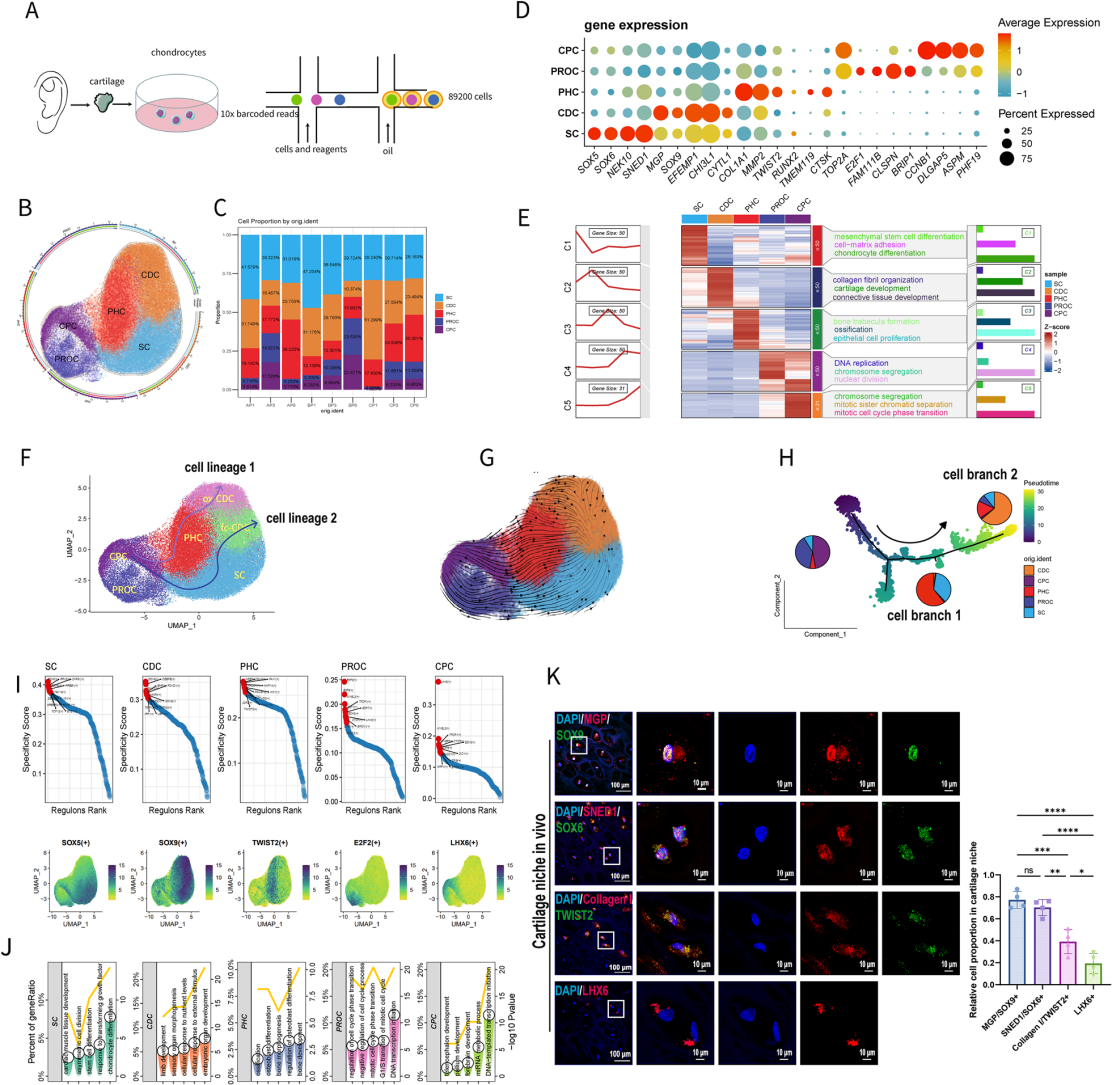
Single cell RNA-seq revealed cell lineages and differentiation trajectories of human chondrocytes. **A** Work flow of single cell RNA-seq. **B** UMAP of 5 distinct cell types (SC, CDC, PHC, PROC, CPC). **C** Cell proportions of 5 cell types in all samples. CDC and SC showed decreasing trend while CPC and PHC showed increasing trend. **D** Dot plot of marker genes in 5 cell types. **E** Heatmap and representative GO terms of 5 cell types. **F** Cell lineages predicted by “Slingshot”. **G** Prediction of cellular dynamics by “sctour”. **H** Differentiation branches predicted by “monocle2” and cell proportions of 3 main states. One trajectory from CPC to PHC exhibited hypertrophic cellular features and another trajectory from CPC, SC to CDC showed cartilage specific cellular features. **I** Top ranked TF activities of each cell type and representative TF feature plots of each cell type. **J** GO terms of TFs enriched in 5 cell types. **K** Immunofluorescence of LHX6 (CPC), SOX6/SNED1 (SC), SOX9/MGP (CDC), TWIST2/Collagen I (PHC) expression in cartilage niche (n = 3 for each group). SC and CDC account for the majority of chondrocyte in vivo. I (Data are presented as means ± SD; Statistical analysis was performed using one-way ANOVA with Tukey’s post hoc test as appropriate; SC: Stromal Cell, CDC: Chondrocyte Differentiated Cell, PHC: Pre-Hypertrophy Cell, PROC: Proliferative Cell, CPC: Chondrocyte Progenitor Cell; ox: oxidative, fc: functional; GO: Gene Ontology; TF, transcription factor; *p < 0.05, **p < 0.01, ***p < 0.001, ****p < 0.0001, ns: no significance)

We obtained auricular cartilage from clinical patients and performed in vitro isolation, expansion, and passaging. Utilizing our previously established cell dedifferentiation model [11, 23], we selected cells from the P1 (1st, primary), P3 (3rd, lower passage), and P6 (6th, higher passage) for single-cell sequencing (Fig. 1A, 3 patients, 3 passages per patient, totaling 9 samples). According to our previous study [11], P1 exhibited abundant collagen II and aggrecan expression, with a significant reduction at P3, culminating in complete loss by P6 and subsequent passages. Thus, P3 represents the onset of early dedifferentiation, whereas P6 reflects the later stages of this process.

Following sequencing and data quality control, transcriptomic data from 89,200 cells were acquired (Figure S1A). Dimensionality reduction and clustering using Seurat V4 [24] revealed five distinct cell types (Fig. 1B) based on highly expressed genes (Fig. 1D, Figure S1B-C): SC (Stromal Cells: *SOX5, SOX6, SNED1, NEK10*), CDC (Chondrocyte Differentiated Cells: *SOX9, MGP, EFEMP1, CHI3L1*), PHC (Pre-Hypertrophy Cells: *COL1A1, MMP2, TWIST2, CTSK*), PROC (Proliferative Cells: *E2F1, FAM11B, CLSPN, BRIP1*) and CPC (Chondrocyte Progenitor Cells: *CCNB1, DLGAP5, ASPM, PHF19*). Cell proportions for SC (blue module) and CDC (orange module) decreased from P1 to P6 while those for PHC (red), PROC (dark blue) and CPC (purple) increased gradually (Fig. 1C). GO (Gene Ontology) terms enriched in these five cell types indicated specific functions for their distinct cell types (Fig. 1E).

We analyzed two distinct cell trajectories from CPC to CDC (slingshot [25]: Fig. 1F; sctour [26]: Fig. 1G; Monocle2 [27]: Fig. 1H; Vector [28]: Figure S1G). CDC can be further subdivided into two subgroups: ox-CDC (oxidative) and fc-CDC (functional) (Figures S1D-E). CytoTrace scores [29] revealed that CPC, with the highest score, exhibited the lowest degree of differentiation, while CDC, with the lowest score, displayed the highest degree of differentiation (Figure S1F). GO terms for differentially expressed genes in these branches highlighted their distinct functions (Figure S1H). Marker genes exhibited varying expression trends throughout differentiation (Figure S1I).

SCENIC [30] analysis identified transcription factors for the 5 cell types (Figure S2A). Based on specificity scores and expression levels (Figure S2B), we identified the most critical TFs (transcription factors) for each cell type (SOX5/SOX6 in SC, SOX9 in CDC, TWIST2 in PHC, E2F2 in PROC, LHX6 in CPC. Figure 1I) and their transcriptional network (Figure S2C). GO terms for these transcription factors revealed functions consistent with those described for the respective cell types (Fig. 1J). According to the CSI (Connection Specificity Index) scores, all transcription factors clustered into four categories (Figure S2D), with Module 1 and Module 3, primarily composed of SC and PHC transcription factors/TFs, showing similar concendistraibutions (Figure S2E), indicating competitive interaction between them. Within the auricular cartilage niche, we validated the expression of marker genes and the proportion of positive cells across the 4 major functional subgroups, with SC and CDC predominating in cell proportions (Fig. 1K).

### Cell passaging experienced various cellular phenotypic changes of chondrocytes

**Fig. 2:**
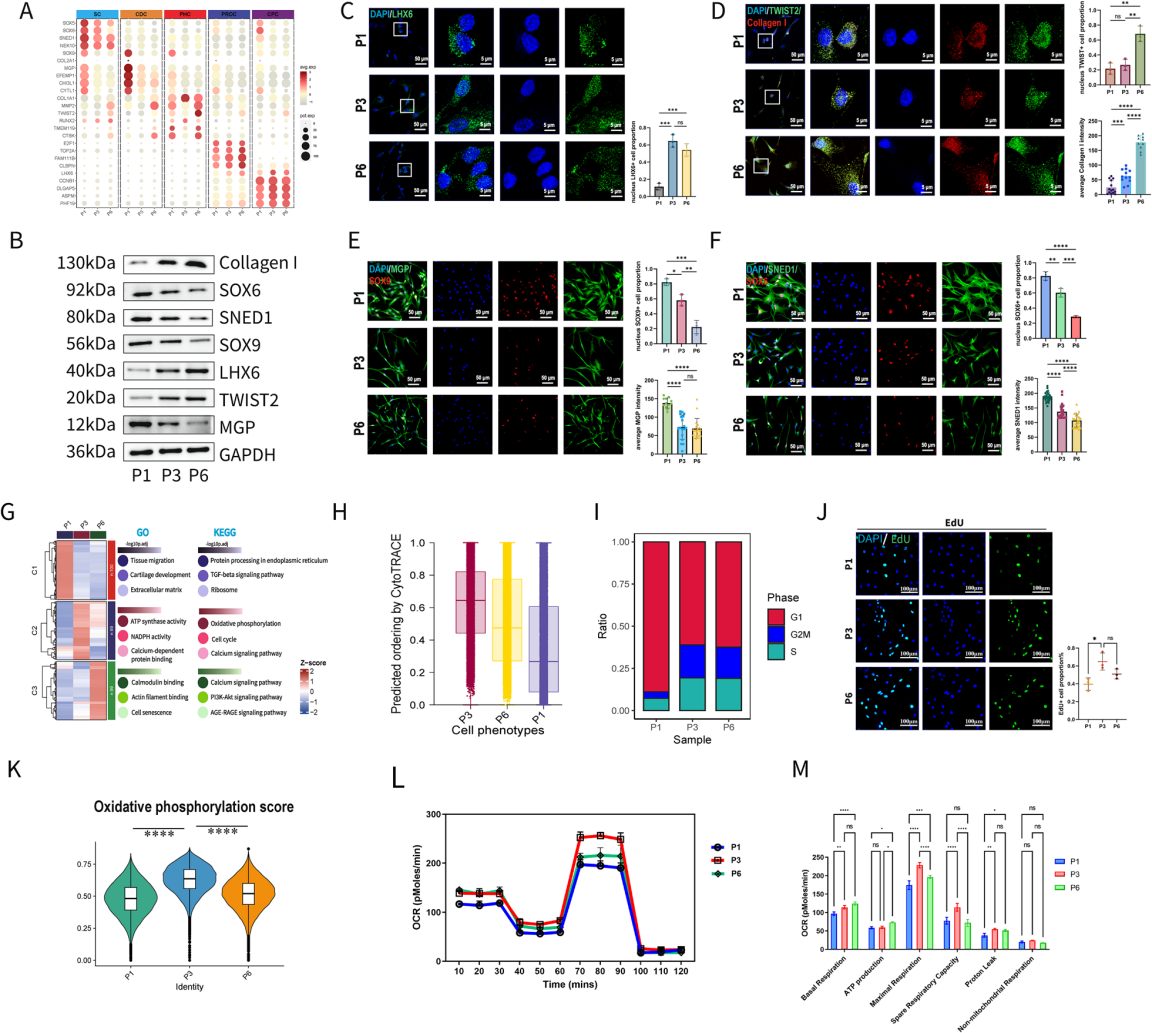
Cell passaging experienced various cellular phenotypic changes. **A** Biomarkers expression in scRNA data among 3 passages. **B** Protein expression levels by WB. **C–F**: LHX6 (**C**), TWIST2/Collagen I (**D**), SOX6/SNED1 (**E**) and SOX9/MGP (**F**) immunofluorescence staining for 3 passages. Biomarkers exhibited similar trends to their proportions. **G** Heatmap, GO and KEGG enrichment analysis of DEGs among 3 passages. **H** Differentiative score measured by “CytoTrace”. More score represents less differentiative degree. **I** Proportions of cell cycle phases. **J** EdU positive cell proportion of 3 cell passages (n = 3 each group). **K** Oxidative phosphorylation score.** L** OCR curves of chondrocytes in 3 passages (n = 3 each group).** M** 6 major parameters of OCR assay for 3 passages. Totally, markers for SC and CDC gradually decreased while those for CPC and PHC increased. P1 cells exhibited more cartilage cellular features. P3 cells for early dedifferentiation exhibited highest aerobic respiration level, proliferative ability and least differentiative degree which were closer to dedifferentiation. P6 cells showed hypertrophic differentiation features. (Data are presented as means ± SD; Statistical analysis was performed using one-way ANOVA with Tukey’s post hoc test as appropriate; DEGs: Differentially Expressed Genes; WB: Western Blot; OCR: Oxygen Consumption Rate; *p < 0.05, **p < 0.01, ***p < 0.001, ****p < 0.0001, ns: no significance)

Over the three passages, the proportions of SC and CDC cells and the expression levels of their marker molecules gradually decreased, whereas those associated with CPC (rapidly from P1 to P3) and PHC (rapidly from P3 to P6) increased. (Fig. 2A–F). Differentially expressed genes (DEGs, Figure S3A) and there GO/KEGG (Kyoto Encyclopedia of Genes and Genomes) enrichment were identified (Fig. 2G). In P3 cells, P3 showed highest “CytoTrace” score and proportions of S and G2/M phases were highest (Fig. 2H, I), correlating with the lowest degree of differentiation and highest proliferative capacity (Fig. 2J).

Cellular metabolic behaviors were obviously significant across cell passages (Fig. 2G, Figure S3B), therefore we performed live-cell metabolic assays to measure the OCR (oxygen consumption rate, representing aerobic respiration) and ECAR (extracellular acidification rate, representing anaerobic respiration). The oxidative phosphorylation gene set score (Figs. 2K) and OCR curves increased from P1 to P3 and declined at P6, with P3 cells showing significantly higher basal respiration, maximal respiration, and spare respiratory capacity (Fig. 2L, M). Conversely, ECAR exhibited an opposite trend, with P3 cells displaying significantly lower glycolysis and glycolytic capacity. (Figure S3C). Conversely, cellular anaerobic respiration demonstrated an opposing trend, with P3 cells exhibiting significantly lower glycolysis and glycolytic capacity (Figures S3C). Thus, an aerobic-to-anaerobic transition occurred during cell passaging.

Cell-chat analysis indicated that overall cellular communications decreased, but FGF signal increased, potentially contributing to PHC differentiation (Figure S3D-I).

Similar changes in gene expression levels and functional analyses of differentially expressed genes were observed in previous bulk RNA transcriptome data (Figure S4A–I). Additionally, cell subpopulations resembling high-passage phenotypes predominantly consisted of PHC (scissor^**+**^), while those related to low-passage phenotypes were primarily composed of SC and CDC (scissor^**−**^; Figure S4J-M).

In summary, we constructed a high-resolution single-cell map of human chondrocytes, elucidating the dedifferentiation lineages of these cells. This map facilitated the identification of new precise biomarkers (decreased MGP and SNED1), for human chondrocyte dedifferentiation. Furthermore, cartilage specific phenotypes continuously decreased (MGP, SOX9, SOX6 and SNED1 expression), P3 cells exhibited more dedifferentiated phenotypes (highest proliferative capacity, aerobic metabolism level, most CPC cell proportion and lowest differentiation degree) and P6 cells, traditionally late dedifferentiation, showed more hypertrophic phenotypes (highest collagen I and MMP2 expression, higher differentiation degree and anaerobic respiration than P3, most PHC cell proportion).

### Cell passaging is accompanied by the gradual opening of chromatin

**Fig. 3:**
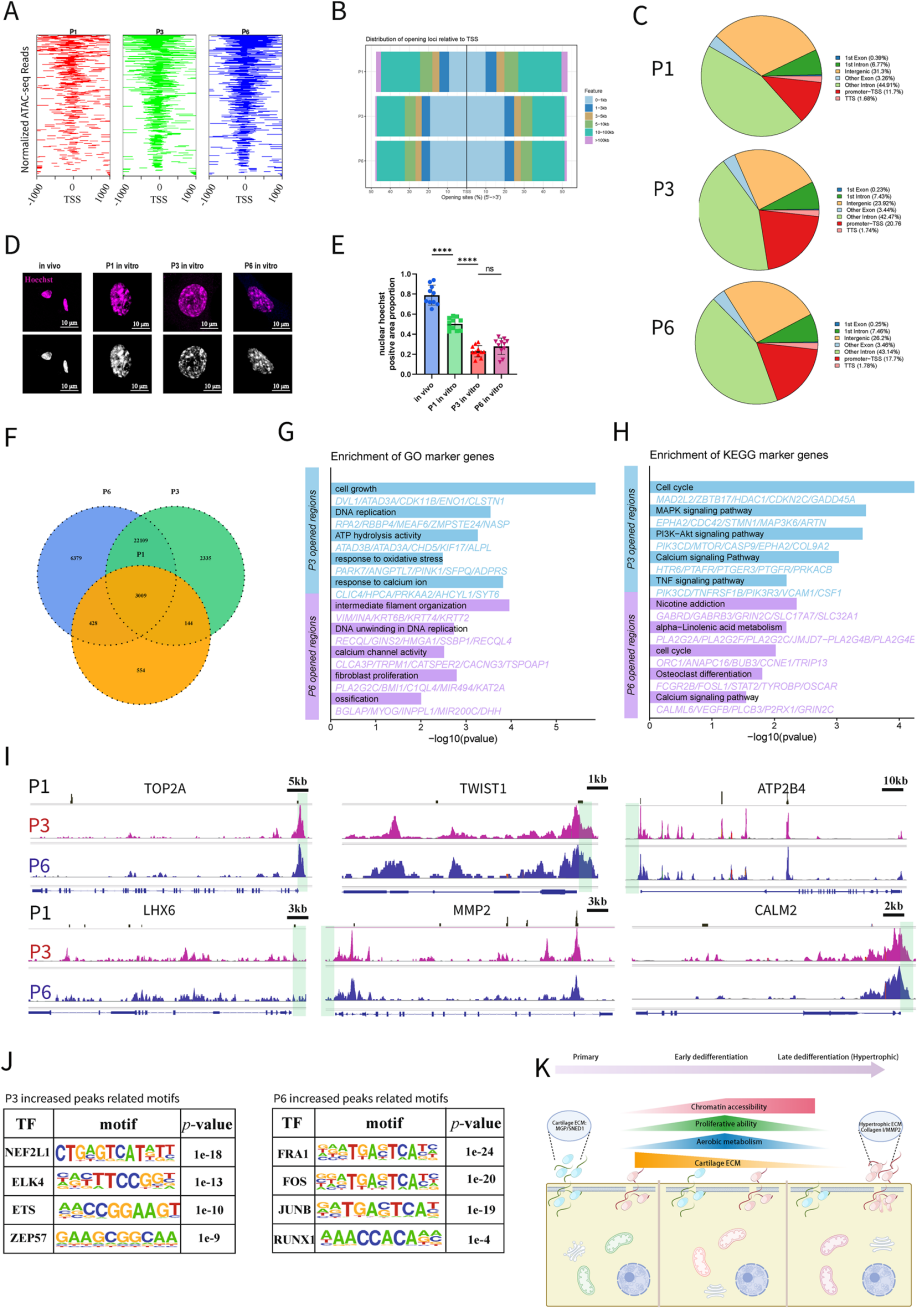
ATAC-seq revealed rapidly increased chromatin accessibility during cell passaging. **A** Detected peaks within 1 kb of TSS in 3 passages. **B** Distribution of opening loci relative to TSS. **C** Distribution features of P1, P3 and P6 specific peaks. **D** Hoechst staining(upper) and its grey-scale(under) of chondrocytes in vivo, P1, P3 and P6 in vitro. **E** Nuclear Hoechst positive proportion of chondrocytes (n = 10 for each group). From P1 to P3, chromatin accessibility increased rapidly. **F** Venn diagram of intersected gene regions opened in P1, P3 and P6 cells. **G** GO terms of increased opening gene regions in P3 and P6 cells. **H** KEGG pathways of increased opening gene regions in P3 and P6 cells. **I** Representative opening gene regions (*TOP2A* and *LHX6* for cell proliferation and stemness, *TWIST1* and *MMP2* for hypertrophy, *ATP2B4* for metabolism, *CALM2* for calcium signal) of 3 passages. Green boxes represent promoter areas.** J** Representative related motifs of increased peaks in P3 and P6 cells. **K** Schematic diagram showed chondrocyte dedifferentiation features from early to late dedifferentiation (Hypertrophy). (TSS, transcription start sites; Data are presented as means ± SD; Statistical analysis was performed using one-way ANOVA and with Tukey’s post hoc test as appropriate. ****p < 0.0001)

Gene expression is closely associated with chromatin accessibility within the nucleus. Therefore, we performed ATAC-seq (Assay for Transposase-Accessible Chromatin using high-throughput sequencing) on P1, P3, and P6 cells. In P3 and P6 cells, we observed an increase in the number of peaks within 1 kb of TSS (transcription start sites) compared to P1 (Fig. 3A, B), indicating greater chromatin accessibility. The increase in peaks was predominantly noted in promoter regions (Fig. 3C). Hoechst staining revealed that areas of dense chromatin gradually decreased with passaging, while chromatin openness increased (Fig. 3D, E).

Peak calling identified open chromatin regions, with 24,444 additional regions in P3 compared to P1, and a further increase of 6379 in P6 (Fig. 3F). The increased open chromatin regions in P3 were primarily linked (Fig. 3G–H) to cellular growth and proliferation (*TOP2A* and *LHX6*), aerobic respiration (*ATP2B4*) and calcium signaling pathway (*CALM2*) The increased open chromatin regions in P6 were associated with calcium pathways and ossification (*TWIST1* and *MMP2*; Fig. 3I). Motifs for TFs associated with oxidative stress and the cell cycle (*NFE2L1, ELK4, ETS, ZEP57*) were enriched in P3 cells, while those linked to cell senescence and ossification (*FRA1, JUNB, FOS, RUNX1*) were enriched in P6 cells (Fig. 3J). The degree of chromatin openness increased rapidly from P1 to P3, providing cells with the potential for multiple differentiation pathways. From P3 to P6, various regulatory elements subsequently bound to the chromatin, allowing continued differentiation into multiple phenotypes. In addition to original chondrocyte characteristics, traits of hypertrophy, ossification, and senescence emerged.

In this segment, we validated our previous findings regarding chromatin accessibility, confirming that specific gene regions associated with cellular characteristics became open.

Furthermore, P3 cells displayed the most robust capability for trilineage induced differentiation and clonal colony formation (Figure S5). Collectively, we verified biomarkers and phenotypic features identified through single-cell analysis, confirming that P3 cells exhibited more dedifferentiated characteristics, while P6 cells displayed hypertrophic features. Here, we summarized the whole process in Fig. 3K.

### Increased calcium influx and reversed P3 gene expression of cartilage specific phenotypes

**Fig. 4:**
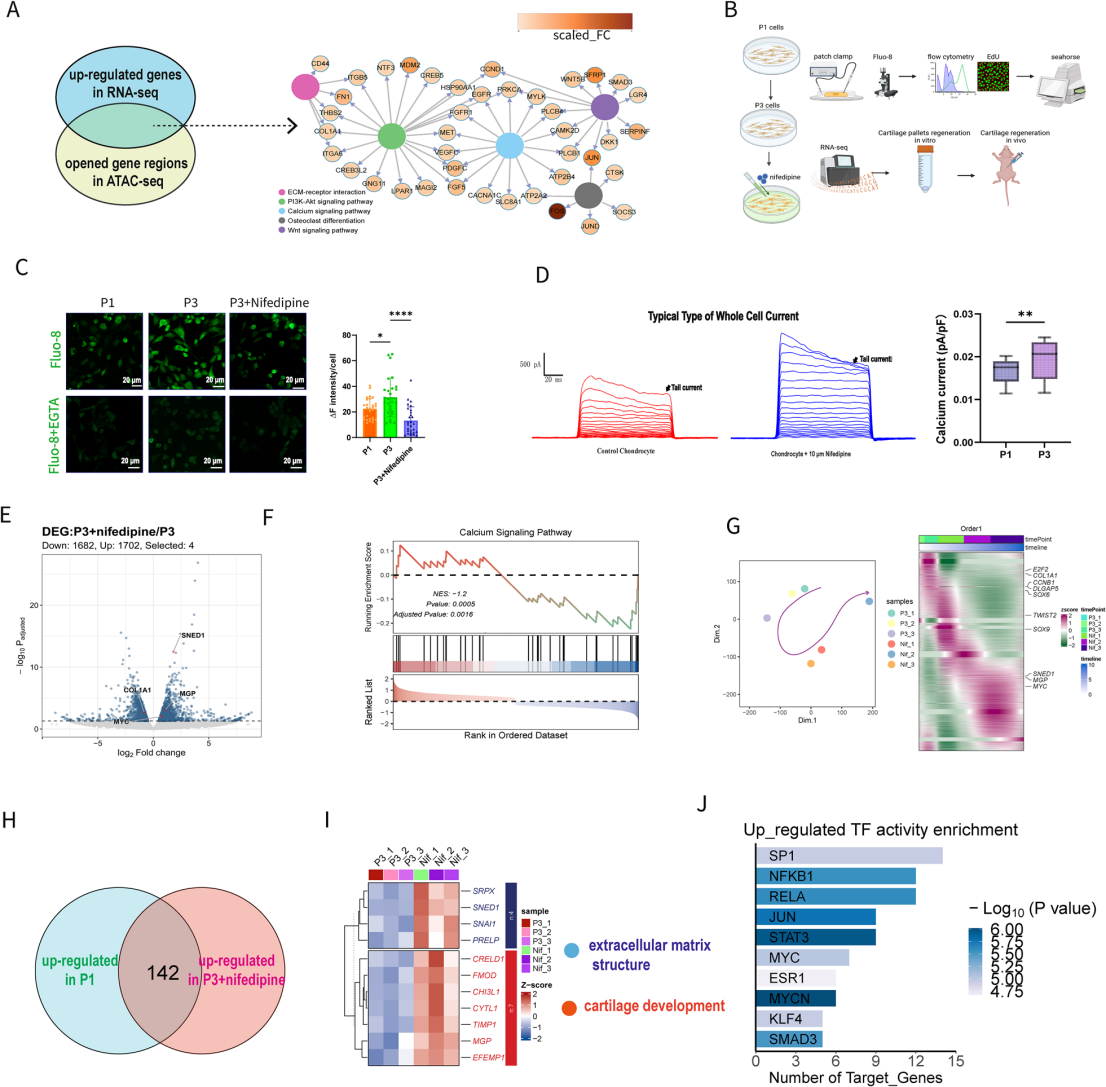
Inhibiting calcium signal by nifedipine showed reversed effect on P3 gene expression by RNA-seq. **A** Gene-pathway interaction network of KEGG analysis for genes intersected by continuous up-regulated in scRNA-seq and opened genes in ATAC-seq. Calcium signaling pathway showed central regulating role. **B** Schematic illustration of research flow for calcium signal in chondrocytes. **C** Intracellular calcium levels (ΔF) measured by Fluo-8 staining in P1, P3, and P3 + nifedipine-treated cells. Nifedipine reduced calcium levels; EGTA was used as a negative control. **D** Typical whole cell current curves of chondrocytes decreased after co-culturing with nifedipine. Decreased current values represent calcium current and P3 cell showed higher calcium current. **E** Volcano plot of DEGs between P3 and P3 + nifedipine cells (adjusted p-value < 0.05, |log2Foldchange|> 1). **F** GSEA analysis exhibited that calcium signal was decreased by nifedipine. **G** Measured differentiation direction driven by nifedipine and gene expression trend along timeline. **H** 142 vital genes were intersected between P1 cell in scRNA and P3 + nifedipine in RNA-seq. **I** Heatmap of representative up-regulated genes. GO terms were marked. **J** Up-regulated TF activity measured based on intersected genes. (Data are presented as means ± SD; Statistical analysis was performed using Student’s t test (**D**) and one-way ANOVA (**C**) and with Tukey’s post hoc test as appropriate GO: Gene Ontology; KEGG: Kyoto Encyclopedia of Genes and Genomes; DEGs, differentially expressed genes; TF, transcription factor; *p < 0.05, **p < 0.01, ***p < 0.001, ****p < 0.0001, ns: no significance)

Next, we aimed to identify key genes and mechanisms driving chondrocyte dedifferentiation. Given that dedifferentiation is a continuous process and ATAC-seq data showed persistently open chromatin regions, genes with both continuously increasing expression and sustained chromatin accessibility were likely to play major regulatory roles. In this intersected gene set, KEGG enrichment showed important regulating role (Fig. 4A). Additionally, genes located in calcium signaling pathway were significant in cell communications (FGF5 in Figure S3I) and more chromatin accessibility (Fig. 3H, I). Notably, among the genes encoding calcium channel proteins, *CACNA1C* exhibited the highest expression level in our single-cell data. *CACNA1C* encodes the L-type calcium channel protein Cav1.2, and its protein expression gradually increased with successive passages (Figure S6A). All these evidences indicated increased calcium influx.

The intracellular calcium ion concentration was assessed (Fig. 4B) using Fluo-8, revealing higher fluorescence intensity in P3 cells compared to P1 cells. The addition of nifedipine, a Cav1.2 inhibitor, resulted in a significant decrease in fluorescence intensity (∆F, Fig. 4C). Whole-cell patch clamp recordings were utilized to measure total cellular currents in chondrocytes before and after nifedipine treatment. Post-treatment, the outward current increased, with the current difference representing the calcium current. In P3, the difference in current values after inhibiting calcium influx was notably greater than in P1, indicating an increase in calcium current (Fig. 4D, Figure S6B).

Following a 48-h culture in medium containing 10 µM nifedipine, RNA-seq was performed on P3 cells (native and nifedipine-treated, each with 3 duplicates). Nifedipine treatment led to the upregulation of 373 genes and downregulation of 200 genes (adjusted *p* value < 0.05, |log2Foldchange|> 1, Fig. 4E). KEGG enrichment analyses revealed decreased calcium signal (Fig. 4F). Based on gene expression along a predicted timeline [31] (Figure S6C), nifedipine may promote chondrogenic differentiation in P3 cells (Fig. 4G). By intersecting with differentially expressed genes in P1, we identified 142 upregulated (Fig. 4H) and 190 downregulated overlapping genes (Figure S6B). The upregulated overlapping genes and TFs were primarily associated with extracellular matrix structure and cartilage development (Fig. 4I, J) while the downregulated genes were linked to cell cycle regulation, stem cell proliferation, calcium transport, and ATP-dependent activities (Figure S6D, E). Therefore, inhibiting calcium influx may prevent the initiation of dedifferentiation, potentially recovering the cellular phenotypes of P3 cells.

### Enhanced cartilage regeneration by blocking calcium signal with nifedipine

**Fig. 5:**
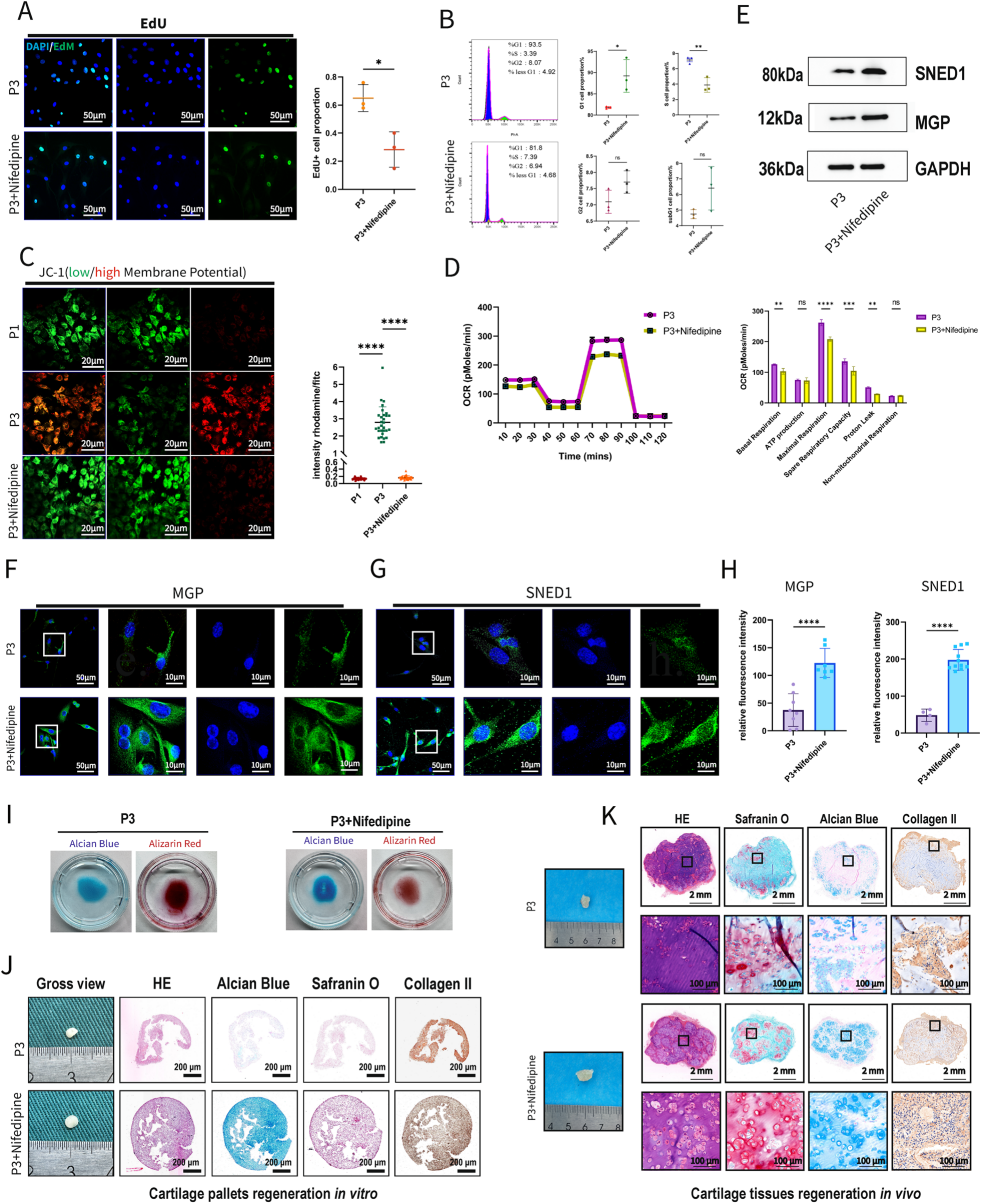
Inhibiting calcium signal by reversed dedifferentiated cellular phenotypes and promoted cartilage regeneration outcomes of P3 cells. **A** EdU staining and positive cell proportions of P3 and P3 + nifedipine cells (n = 3 each group). **B** Difference of cell cycle phases by flow cytometry (n = 3 for each group). Nifedipine reversed cell proliferative capacity. **C** JC-1 staining of P1, P3 and P3 + nifedipine cells (left). Difference of rhodamine/fitc intensity proportion among 3 groups (right) (n = 30 each group). **D** OCR curves and difference of 6 major parameters (n = 3 each group. Nifedipine reversed aerobic respiration of P3 cells. **E** MGP and SNED1 protein expression difference. F-G Fluorescence staining of MGP and SNED1. **H** Fluorescence intensity difference of MGP and SNED1 (n = 7 each group). Nifedipine promoted cartilage ECM by reversing MGP and SNED1 expression. **I** Alcian blue and alizarin red staining after undergoing chondrogenic induction and osteogenic induction for 14 days. **J** Gross view, HE, Alcian Blue, Safranin O and Collagen II staining of 21-days induced cartilage pallets in vitro from P3 and P3 + nifedipine cells. **K** Induced cartilage tissues in vivo from P3 and P3 + nifedipine cells. Nifedipine promoted cartilage regeneration of P3 cells. (Data are presented as means ± SD; Statistical analysis was performed using Student’s t test (**A**, **B**, **D**, **H**) and one-way ANOVA (**C**) and with Tukey’s post hoc test as appropriate; OCR: Oxygen Consumption Rate; HE: Hematoxylin and Eosin; *p < 0.05, **p < 0.01, ***p < 0.001, ****p < 0.0001, ns: no significance)

Based on the RNA-seq, we next investigated whether inhibiting calcium influx could restore cellular phenotypes and promote cartilage regeneration. After co-culturing P3 cells with nifedipine for 48 h, the proportion of EdU^+^ cells significantly decreased (Fig. 5A). The proportion of cells in the G1 phase increased, while the proportion in the S phase decreased (Fig. 5B), indicating that nifedipine inhibits the proliferation of P3 cells.

Changes in mitochondrial membrane potential were assessed using JC-1 dye. Compared to P1 cells, P3 cells exhibited significantly higher rhodamine fluorescence intensity relative to FITC fluorescence (intensity rhodamine/FITC > 1). Nifedipine treatment significantly reduced rhodamine fluorescence intensity (Fig. 5C). P3 cells co-cultured with nifedipine showed significantly lower basal respiration, maximal respiration, and spare respiratory capacity (Fig. 5D). These findings demonstrate that inhibiting calcium influx can reduce aerobic metabolism in P3 cells.

Additionally, Western blot results showed increased protein levels of MGP and SNED1 (Fig. 5E). Fluorescent staining confirmed these results, indicating a recovery of P3 cellular phenotypes (Fig. 5H). PCR results showed increased mRNA levels of significant TF genes (SOX5, SOX6 and SOX9, Figure S6F). After inducing P3 cells with a chondrogenic medium containing 10 µM nifedipine for 2 weeks, Alcian blue staining was significantly enhanced. Conversely, induction with the same concentration of nifedipine in an osteogenic medium for 2 weeks resulted in significantly diminished alizarin red staining (Fig. 5I). Following in vitro cartilage regeneration with a chondrogenic medium containing nifedipine and in vivo regeneration via GelMA (Gelatin Methacryloyl) loaded with nifedipine, the regenerated cartilage tissues exhibited a more mature structure (as shown by HE staining) and increased extracellular matrix (as indicated by Alcian Blue and Safranin O staining). However, no significant difference was observed in the content of type II collagen (Fig. 5J, K, Figure S6G). Inhibiting calcium influx reversed cellular phenotypes and enhanced cartilage formation, suggesting a restoration of dedifferentiated phenotypes.

### Calcium signal drives early differentiation by inhibiting *MYC* expression

**Fig. 6:**
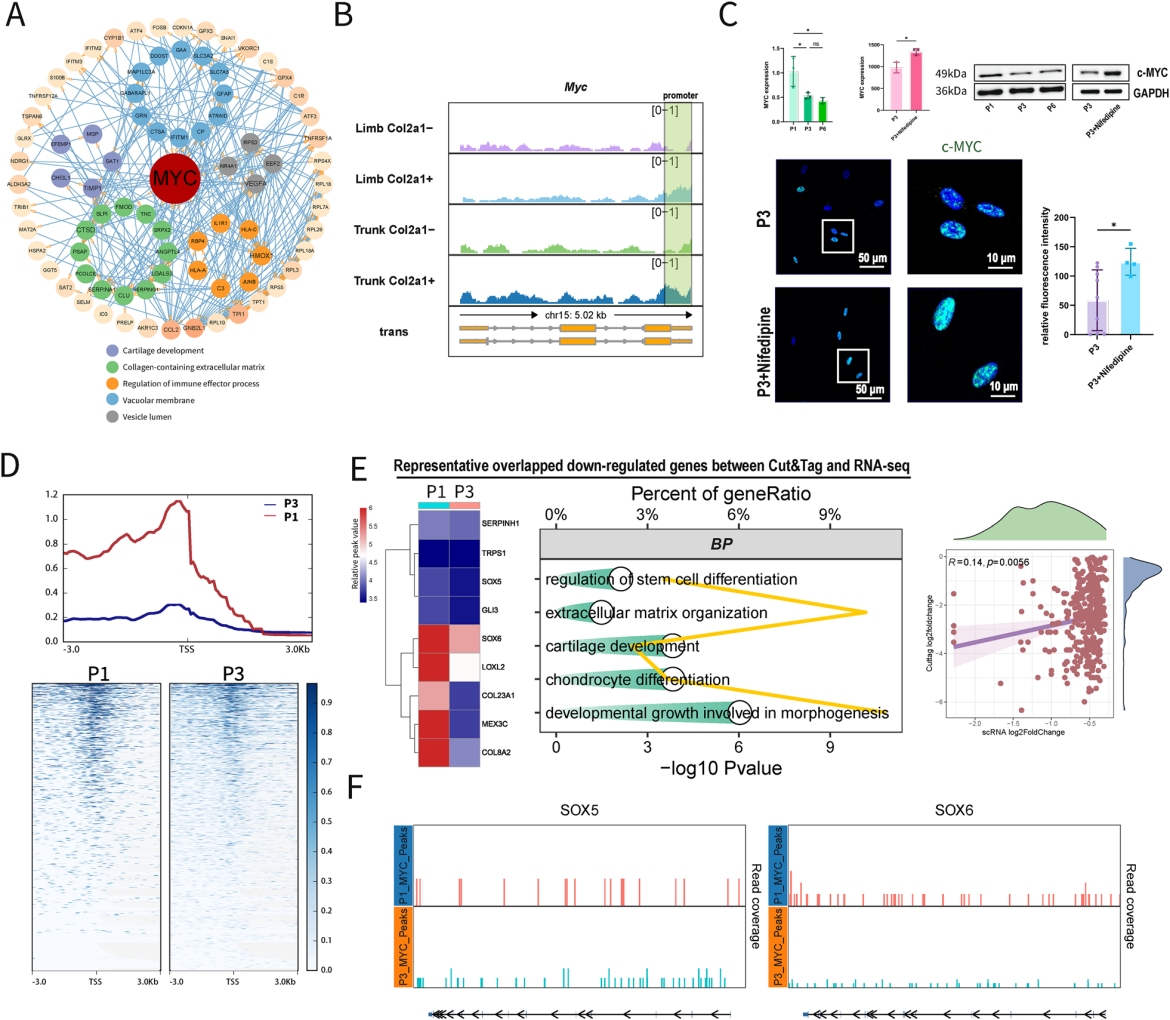
Calcium signal targets *MYC* to regulate chondrocyte phenotypes. **A** PPI network of vital genes identified by RNA-seq revealed central regulatory role of MYC in chondrocytes. **B** MYC expression difference across cell passages and after adding nifedipine. MYC decreased by cell passaging and reversed by nifedipine.** C** Immunofluorescence of c-MYC of P3 and P3 + nifedipine (n = 5 each group). **D** Identified peaks within 3 kb of TSS of c-MYC cut &tag in P1 and P3 cells. MYC regulatory effect were significantly decreased in P3 cells. **E** Representative overlapped gene regions of cut&tag and scRNA and their GO terms. Relation of log2foldchange of down-regulated genes in P3 cells. MYC can regulate cartilage ECM-related gene expression. F Representative detected open gene regions of *SOX5* and *SOX6*. (Data are presented as means ± SD; Statistical analysis was performed using Student’s t test (**A**, **B**) and one-way ANOVA (**A**) and with Tukey’s post hoc test as appropriate. *p < 0.05, **p < 0.01, ***p < 0.001, ****p < 0.0001, ns: no significance)

Regarding the signaling mechanism, we tried to identify key targeting molecules of calcium signal. Based on the PPI network constructed by vital intersected genes (Fig. 6A), considering the upregulated TF (Fig. 4J) activity and the gene-biological-process network, MYC was identified as the most critical TF. In single-cell data, *MYC* expression significantly decreased across all five cell subgroups in P3 cells (Figure S7A). A similar trend was observed in mouse chondrocytes [17] (Figure S7B). Within the transcriptional regulatory network of MYC calculated by SCENIC, MYC was shown to regulate the expression of cartilage-related specific genes (Figure S7C). Concurrently, *MYC* expression was positively correlated with *MGP* and *SNED1* in nifedipine-related RNA-seq data (Figure S7C). The gene sets in RNA-seq associated with MYC exhibited positive correlation with extracellular matrix secretion (Figure S7D). In mouse H3K27ac ChIP-seq analysis, we observed significantly higher binding signals in col2a1 + cells of both the trunk and limbs, indicating that Myc exhibits highly specific transcriptional regulatory activity in chondrocytes (Fig. 6B). Moreover, Both RNA and protein (c-MYC) expression levels were detected and recovered (Fig. 6C) following calcium influx inhibition.

Increased intracellular calcium concentration upregulated CAML1 and CALM2 (Figures S7E–F), encoding calmodulin, which may interact with HuR, a major RNA binding protein for MYC; blocking calcium influx increased MYC RNA expression that binds with HuR, suggesting calmodulin could competitively bind HuR and promote MYC degradation (Figures S7G–I).

We next employed cut&tag to explore regulatory targets of c-MYC in P1 and P3 cells. The detected peaks in P3 were fewer than those in P1 near the TSS (Fig. 6D), indicating a weaker transcriptional effect. The reduction in peaks primarily occurred in promoter regions (Figure S7J), with the c-MYC matching motif detected (Figure S7K). GO terms for downregulated overlapping genes were mainly related to extracellular matrix and chondrocyte differentiation. The downregulated fold change from peak values in cut&tag and gene expression in scRNA-seq data were positively correlated (Fig. 6E). Notably, *SOX5* and *SOX6* (key regulatory TFs for SC) were regulated by c-MYC, with decreased peaks in these gene regions in P3 cells potentially resulting in ECM loss (Fig. 6F).

**Fig. 7:**
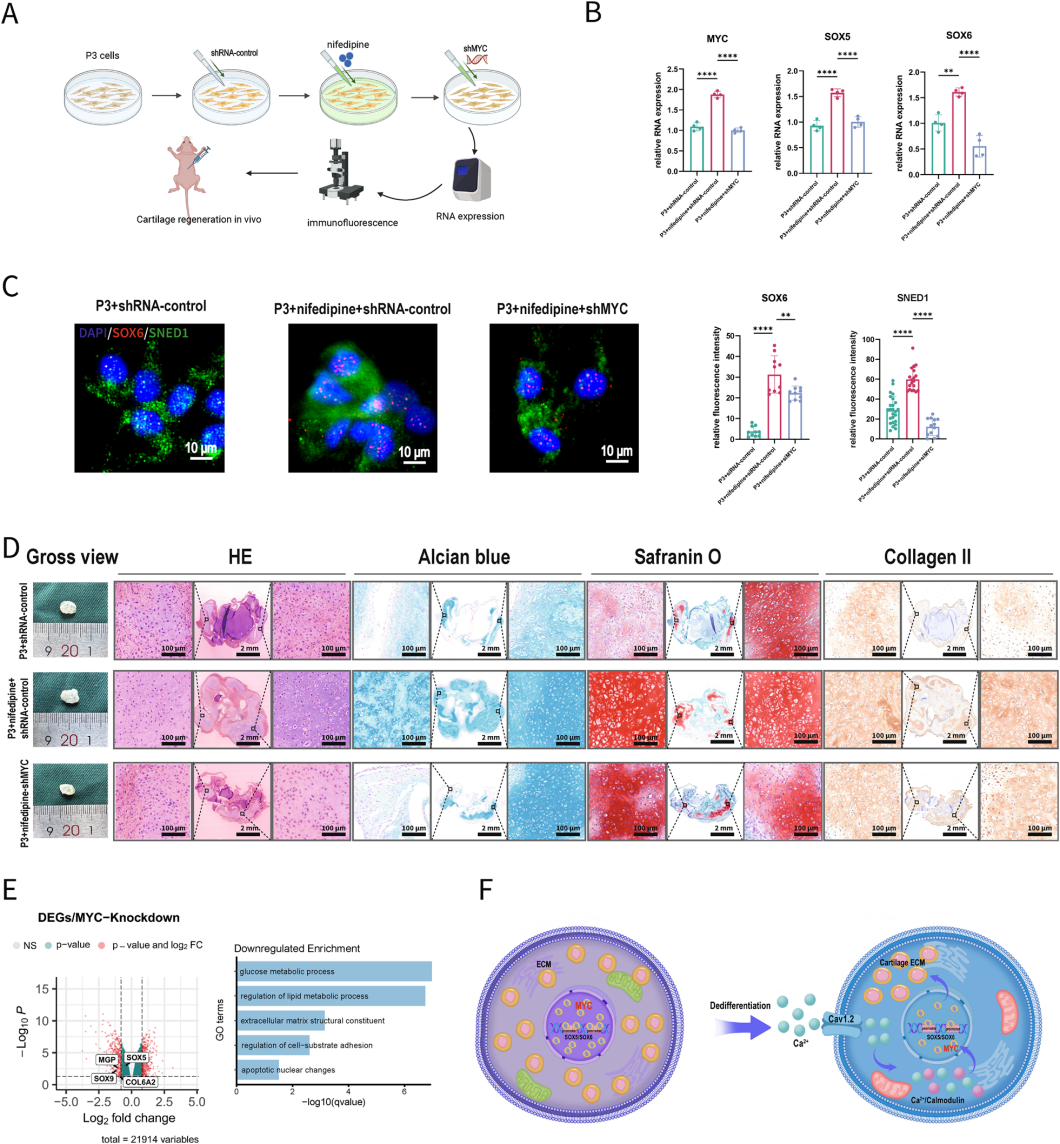
Decreased MYC expression by calcium signal can hinder cartilage ECM level and regeneration in vivo. **A** Schematic illustration of the effect of *MYC* expression on cartilage regeneration. **B** In P3 cells, increased RNA expression levels of *SOX5/SOX6* resulting from inhibiting calcium influx were reversed by MYC knockdown (n = 4 for each group). **C** Reversed SOX6 and SNED1 immunofluorescence intensity. **D** In P3 cells, better outcome of induced cartilage tissues resulting from inhibiting calcium influx were reversed by MYC knockdown in vivo. **E** Schematic diagram of the role of calcium-MYC-SOX5/SOX6 axis in the early dedifferentiation and cartilage regeneration of chondrocytes. (ECM: Extracellular matrix; Data are presented as means ± SD; Statistical analysis was performed using one-way ANOVA and with Tukey’s post hoc test as appropriate. *p < 0.05, **p < 0.01, ***p < 0.001, ****p < 0.0001, ns: no significance)

Following MYC knockdown by shRNA in P3 cells (Fig. 7A), the upregulation of gene expression induced by calcium signal inhibition was reversed, confirming the regulatory targets of c-MYC (Fig. 7B, C). In P3 cells, better-induced cartilage tissues resulting from calcium influx inhibition were reversed by MYC knockdown in vivo (Fig. 7D). Furthermore, when MYC was knocked down in stem cells, the expression of genes such as SOX9 and SOX5 was reduced, accompanied by a downregulation of critical functions related to extracellular matrix secretion (Fig. 7E). These results suggest that MYC, as a target of calcium signaling, can influence the extracellular matrix levels in chondrocytes by upregulating the cartilage-related transcription factors, thereby reversing dedifferentiation and improving cartilage regeneration outcomes (Fig. 7F).

## Discussion and conclusion

Understanding the biological processes and mechanisms underlying phenotypic changes in chondrocyte dedifferentiation is crucial for advancing the clinical application of tissue-engineered cartilage. In this study, we first constructed a high-resolution map and identified biomarkers of human chondrocyte dedifferentiation.

We delineated five distinct chondrocyte cell types, each defined by a specific marker gene set. SOX5, SOX6 [32–35] and SNED1 [36, 37] were associated with ECM secretion [38] and the inhibition of hypertrophic differentiation in chondrocytes [39]. SOX9 [40, 41] and MGP [42] were crucial for chondrocyte differentiation [43, 44]. Conversely, TWIST2 [45–48], COL1A1 and MMP2 [49–53] were markers of PHC. These all genes have been reported to be related to hypertrophy. PHC exhibits strong FGF signaling, which is often linked to cell proliferation, likely due to its differentiation from PROC/CPC. Additionally, LHX6 [54–56] was identified for progenitor cells, while E2F2 and CLSPN were linked to proliferative cells. Our single-cell study, based on the largest dataset to date, identified precise biomarkers of dedifferentiation, providing a more comprehensive characterization of chondrocyte cell types compared to mouse data [17]. Notably, the loss of *SOX5, SOX6, SNED1* and *MGP* serves as more accurate markers of chondrocyte dedifferentiation than traditional markers like *COL2A1* or *ACAN*.

Regarding cell lineages, chondrocytes originate from CPCs, marking the initial differentiation point. Notably, PHC and SC exhibit a competitive antagonistic relationship during dedifferentiation. An imbalance in this competition during expansion is posited to be a primary factor contributing to the loss of chondrocyte-specific phenotypes, potentially leading to suboptimal outcomes in long-term cartilage tissue engineering.

In previous studies, cells at early dedifferentiated stages can regain cartilage-specific phenotypes, whereas late dedifferentiated cells are more resistant to reversal [17, 21, 22, 57]. This phenomenon has not been properly explained so far. By integrating differential analyses of single-cell RNA and chromatin accessibility data, we concluded that during the early stages of in vitro culture (P3), chondrocytes undergo significant dedifferentiation, losing critical traits such as ECM secretion and reverting to a more primitive state characterized by increased proliferative capacity and rapid chromatin opening. P3 cells exhibited the highest level of aerobic metabolism, endowing them with the potential for multilineage differentiation. As culture progresses to later passages (P6, traditionally considered later dedifferentiation), chondrocytes increasingly differentiate along an alternative trajectory towards hypertrophic chondrocytes, acquiring characteristics typical of ossification and fibrosis. This pattern provides a more comprehensive explanation for the observed phenotypic changes than simple dedifferentiation alone, elucidating that later dedifferentiated cells cannot be restored. Consequently, this suggests that constructing tissue-engineered cartilage necessitates tailored interventions for the distinct phenotypic characteristics of chondrocytes at various passages to achieve optimal performance.

Concerning the signaling pathways driving these phenotypic changes, we propose that the calcium signaling pathway centrally regulates various biological processes. This pathway initiates with the influx of calcium ions, a critical second messenger that subsequently stimulates the upregulation of calmodulin and calmodulin-dependent kinases [58]. This activation cascades into downstream biological alterations. It is established that elevated intracellular calcium concentrations can induce hypertrophy, diminish autophagy, and modulate chondrocyte maturation [59–63]. Furthermore, increased calcium levels may promote chondrocyte calcification [64]. Confirming the augmented influx of calcium ions, we demonstrated that the calcium channel blocker nifedipine effectively reduces this influx and enhances cartilage regeneration.

However, the mechanism by which calcium signaling influences chondrocyte phenotype remains unclear. Calcium influx regulates chondrocyte phenotype through multiple pathways, including the calcineurin–NFAT axis [65, 66], CaMKII activation [67], and PKC [68], MAPK, and PI3K/AKT signaling. However, our RNA-seq analysis after calcium influx blockade revealed no significant changes in NFAT or CaMKII-related gene expression, whereas MYC was prominently upregulated and identified as a key regulatory hub. Based on these findings, we focused our subsequent studies on the Ca^2^⁺–MYC signaling axis. Our findings indicate that calcium signaling downregulates *MYC* expression, subsequently reducing the expression of downstream transcription factors associated with the chondrocyte matrix, like SOX5 and SOX6, thereby driving dedifferentiation. MYC has been recognized for its significant role in tumor proliferation and its important effects on chondrocyte proliferation [69] and differentiation [70–73]. The precise mechanism by which MYC promotes chondrocyte differentiation remains elusive, as previous research has not reached a consensus. Here, we present evidence that MYC, as a chondrocyte-related transcription factor (TF), can enhance ECM secretion capability by upregulating the expression of cartilage-related genes.

Thus, we summarize the role of the calcium-MYC-SOX5/SOX6 signaling axis in early chondrocyte dedifferentiation. We believe that our conclusions are applicable to in vitro dedifferentiation of various chondrocyte types for three main reasons: (1) the metabolic shift from a hypoxic to a normoxic environment; (2) the use of monolayer expansion culture; and (3) the previously reported roles of calcium signaling and MYC in other chondrocyte types as previously mentioned. However, due to limitations in sample availability and ethical considerations, further experimental validation across different chondrocyte types is required to confirm these findings.

In addition, these features resemble those seen in pathological chondrocyte hypertrophy in OA (osteoarthritis) [74–77], (1) decreased extracellular matrix secretion, (2) increased matrix degradation, (3) altered metabolic activity, and (4) enhanced mineralization and ossification. Hence, the chondrocyte dedifferentiation model provides a valuable tool for exploring the mechanisms underlying pathological hypertrophy of articular chondrocytes within their native microenvironment.

Based on these findings, we propose several strategies for clinical celltherapy. First, we aim to regenerate auricular cartilage for children with microtia using tissue-engineered scaffolds loaded with calcium signaling inhibitors. Second, incorporating calcium channel inhibitors into ACI (autologous chondrocyte implantation) strategies may improve the quality of regenerated cartilage in OA treatment. Moreover, exploring whether targeted delivery of calcium signaling inhibitors can improve repair outcomes in animal models of growth plate defects is warranted.

In addition to calcium signaling inhibitors, therapeutic strategies targeting MYC or its downstream effectors may also be developed. However, due to the oncogenic potential of MYC overexpression [78], direct upregulation raises significant safety concerns. Therefore, future studies will focus on identifying safer and more suitable MYC downstream targets for cell-based therapies, with validation in large animal models or organoid systems to enhance clinical translatability.

Furthermore, a clear consensus on seeding density and passage time for cell culture remains lacking, undoubtedly impacting cell phenotypes. Our research was conducted based on established expansion practices within our research group. Different expansion protocols may introduce slight variations in conclusions across cell passages; however, the overall trends should remain consistent. This study provides a foundation for improving chondrocyte culture systems and accurately designing scaffolds, ultimately advancing cartilage regeneration. Targeting calcium signaling and MYC represents an important research direction for our team in the future.

## Methods and materials

### Isolation, culture, expansion and cryopreservation of chondrocytes

Cartilage tissues were obtained from residual ear cartilage collected from patients undergoing tympanoplasty at the Eye and ENT Hospital of Fudan University. Isolation, culture, expansion, and cryopreservation of chondrocytes were performed as previously described [11, 23]. Briefly, cartilage tissue in each specimen was carefully dissected to remove any surrounding fibrous tissue. To isolate chondrocytes, the cartilage specimens were minced into small fragments and digested with 0.15% collagenase (Sigma-Aldrich, St. Louis, MO, USA). Cells were initially seeded at a density of 1 × 10^6^ cells per 10 cm dish in Dulbecco’s Modified Eagle’s Medium (DMEM; HyClone Laboratories, Utah, USA) supplemented with 10% fetal bovine serum (FBS; HyClone Laboratories, Victoria, Australia), with subsequent passages increasing at a threefold increment. High-glucose DMEM medium contains 200 mg/L CaCl₂, according to the manufacturer's information. For calcium inhibition, 10 μmol/L final concentration of nifedipine was added into medium.

### Single cell RNA sequencing

Chondrocytes from passages P1, P3, and P6 were selected for single-cell sequencing. Cells were digested into single-cell suspensions and mixed with 10 × barcodes and Master Mix (10 × Genomics Chromium, Capital Biotechnology, Beijing, China). Single-cell libraries were constructed according to the manufacturer's instructions. High-throughput sequencing was performed on the Illumina NovaSeq platform.

**Table:** 

Samples	Disease	Age (years)	Gender
Patient 1	Otitis media	15	Male
Patient 2	Otitis media	22	Female
Patient 3	Otitis media	10	Male

### Single cell transcriptome data quantifications and analysis

Raw reads were processed through an internal pipeline to generate gene expression profiles. Briefly, cell barcodes and unique molecular identifiers (UMIs) were extracted after filtering reads lacking multiple T-tails. Read2 contains RNA sequences. Cell Ranger uses STAR (v2.7.11) to align Read2 to the reference genome GRCh38 and selects uniquely matched sequences for subsequent analysis. Reads with the same cell barcode, UMI, and gene were combined to calculate the number of UMIs per gene per cell. The UMI count table for each cell barcode was used for further analysis.

The R package Seurat (v4.2.0) was used to analyze sequencing data. After "LogNormalize," "FindVariableFeatures," and "RunPCA," "Harmony" was performed to mitigate batch effects among samples. Subsequently, functions such as "FindNeighbors," "FindClusters," and "RunUMAP" were utilized to visualize all cell types on a two-dimensional map.

The "FindMarkers" function was used to identify differentially expressed genes between samples or cell types. GO and KEGG enrichment analyses were performed using the R package clusterProfiler (v4.9.1).

Cell differentiation trajectories were analyzed using the R packages Monocle2 (v2.29.0), Slingshot (v2.9.0), and sctour (v1.0.0). The degree of cell differentiation was measured using the R package CytoTRACE (v0.3.3). Transcription factor activities were identified using SCENIC (v1.3.1). Cell communications were analyzed using the R package CellChat (v1.6.1).

### Transposase-accessible chromatin with high throughput sequencing (ATAC-seq)

Chondrocytes from passages P1, P3, and P6 were selected for ATAC-seq. Cells were washed with PBS, lysed in chilled buffer, and centrifuged to collect nuclei. Nuclei were treated with transposase, followed by DNA purification and amplification to construct the library. After data quality control using FastQC (v0.11.2), cleaned raw data were aligned to GRCh38. ATAC-seq peak calling was performed using MACS2 (v3.0.1), processing aligned sequencing reads to identify genomic locations indicative of open chromatin. Peaks were annotated with their genomic locations and associated gene information using HOMER (v1.5.2). ATAC-seq experiments were supported by KCSEQHEALTH (Wuhan, China).

### Immunoblotting, immunofluorescence, real-time PCR and in vitro cartilage regeneration.

All protocols were performed as previously described [23]. For immunofluorescence, chondrocytes were fixed on glass slides and washed with phosphate-buffered saline (PBS; Sigma-Aldrich, St. Louis, MO, USA). Cells were incubated overnight at 4 °C with primary antibody, followed by incubation with Alexa Fluor 488/555-conjugated secondary antibodies (Invitrogen, Carlsbad, CA, USA) for 1 h at room temperature in the dark. Nuclei were counterstained with DAPI (Invitrogen). Fluorescence images were acquired using a confocal laser scanning microscope (Zeiss, USA).

For immunoblotting, chondrocytes were lysed in RIPA buffer (Sigma-Aldrich, St. Louis, MO, USA) supplemented with protease inhibitors. Protein samples were separated by SDS-PAGE, transferred onto PVDF membranes (Beyotime, Shanghai, China), and blocked. Membranes were incubated overnight at 4 °C with primary antibodies, followed by incubation with HRP-conjugated secondary antibodies. Bands were visualized using enhanced chemiluminescence and imaged with GeneSys software.

For real-time PCR, chondrocytes (2 × 10^6^ cells per passage) were harvested for total RNA extraction and reverse transcription into single-stranded cDNA. Quantitative real-time PCR was performed using SYBR Green chemistry (MX3000P, Stratagene), with GAPDH serving as the internal control for mRNA normalization.

All antibodies: MGP, TWIST2, SOX6, Collagen I, GAPDH (Proteintech, California, USA); SNED1 (CUSABIO, Wuhan, China); LHX6 (Santa Cruz Biotechnology, Dallas, TX, USA); SOX9 (Abcam, Massachusetts, USA). Primer sequence 5′—> 3′: *MYC*: GGCTCCTGGCAAAAGGTCA—CTGCGTAGTTGTGCTGATGT; *SOX5*: GCCTGGTGGATGGCAAAAAG—CAGTGGCAATGGGGATCTGT; *SOX6*: AGCCTGTAAAGTCCCCAACG—TCCTGATCCCCAAAAAGGGC.

For in vitro cartilage regeneration, chondrocytes from different groups were harvested and centrifuged at 4 × 10^5^ cells per 1 mL of regular culture medium to form pellets. The pellets were then cultured in chondrogenic medium (DMEM with 1% ITS, R&D Systems, Minne) for 3 weeks, followed by gross observation, histological, and immunohistochemical analyses.

Bulk RNA-seq for stem cell was downloaded from GEO database (GSE57538) [79].

### Bulk-RNA sequencing

Chondrocytes were lysed in TRIzol reagent (Sigma-Aldrich, St. Louis, MO, USA) for RNA preservation, followed by extraction with chloroform and precipitation with isopropyl alcohol. RNA pellets were washed twice with 70% ethanol, air-dried, and dissolved in RNase-free water. RNA quantity and quality were assessed using a NanoDrop ND-1000 spectrophotometer. For each group, 1.5 μg of total RNA was used for transcriptome sequencing on the Illumina NovaSeq 6000 platform.

Image processing and base calling were performed using Solexa pipeline software. Sequencing quality of trimmed reads was assessed with FastQC, and reads were aligned to the reference genome using HISAT2. Transcript abundance was estimated using StringTie based on an official gene annotation database to generate transcriptome expression profiles. Differentially expressed genes (DEGs) were identified using the DESeq2 R package through normalization and calculation of fold changes and *p* values.

### Animal experimental design and in vivo cartilage regeneration

Male nude mice (4–6 weeks of age) were obtained from Jiangsu GemPharmatech Company and maintained under standard conditions with a 12 h light/dark cycle and ad libitum access to food and water. Animals that died during the experiment, exhibited technical errors, or had missing critical data points were excluded from the analysis. All procedures adhered to the guidelines for laboratory animal care outlined in the NIH Guide for the Care and Use of Laboratory Animals and were approved by the Institutional Animal Care and Use Committee of the Eye & ENT Hospital of Fudan University (approval number: 2017043).

Sample size calculation was performed using G*Power. The primary outcomes assessed were staining levels of Alcian blue, Safranin O and collagen II. Based on our preliminary data, the calculated effect size (Cohen’s d) was 2.0. Using α = 0.05 and power = 0.8, the minimum sample size required was n = 4 per group. The study was reported in accordance with the ARRIVE guidelines 2.0.

Male nude mice were randomly allocated to either two groups (P3 cell group, n = 4; P3 + nifedipine group, n = 4) or three groups (P3 + shRNA-control, n = 4; P3 + nifedipine + shRNA-control, n = 4; P3 + nifedipine + shMYC, n = 4).

For in vivo cartilage regeneration, P3 cells were suspended in a mixture of 5% (w/v) GelMA and 2.5 mg/mL LAP (lithium phenyl-2,4,6-trimethylbenzoylphosphinate) at a final concentration of 1 × 10^7^ cells/mL. The cell-laden hydrogel was subsequently crosslinked via exposure to ultraviolet (UV) light to form stable cell–hydrogel composites. These composites were subcutaneously injected into the dorsal region of nude mice to establish the in vivo cartilage regeneration model. After 8/12 weeks, the regenerated cartilage tissues were harvested for gross observation and immunohistochemical analysis.

Due to experimental constraints, the experimenters were aware of group allocations during the study. However, outcome assessment and data analysis were conducted in a blinded manner to minimize bias.

Initial subcutaneous injection did not require anesthesia. Eight weeks later, cartilage retrieval was performed under isoflurane anesthesia (2–3%, 1 L/min). Mice remained alive throughout the duration of the experiment.

### Immunohistochemistry

Cartilage tissues collected from nude mice were fixed, embedded in paraffin, and sectioned at 5 μm thickness. Sections were stained with hematoxylin and eosin (H&E), Safranin O/Fast Green (SO/FG), Alcian blue and immunostained for collagen II (Proteintech). Relative protein expression was quantified by calculating the average optical density (AOD) using ImageJ software.

### Oxygen consumption rate and extracellular acidification rate

The OCR and ECAR of chondrocytes were measured in Seahorse extracellular flux analyzer (XFe24, Agilent Technologies). Initially, cells were cultured and allowed to adhere to the surface. Subsequently, media was refreshed and specific concentrations of respiratory chain inhibitors were added into subsequent assays. For OCR, 1 μmol·L^−1^ oligomycin, 1 μmol·L^−1^ FCCP and 1 μmol·L^−1^ antimycin were added. For ECAR, 100 mmol·L^−1^ glucose, 100 μmol·L^−1^ oligomycin and 500 mmol·L^−1^ 2–2-deoxyglucose (2-DG) for ECAR. Three replicates for each condition were used. All testing process were followed by standard protocols (https://www.agilent.com).

### Whole cell patch clamp and calcium ion fluorescent probe

Cells were seeded at a density of 1000 per well in a 24-well plate containing built-in cell-climbing slides. After 2 h of adherence, cells were transferred onto recording slides. Under a 60 × water immersion objective, chondrocytes with clear boundaries and good condition were selected for patch-clamp recording at room temperature. Using a micromanipulator (HEKA, Landenburg, Germany), the electrode position was adjusted under the microscope, applying slight positive pressure before immersion. The electrode was slowly lowered onto the target cell, forming a visible dimple upon gentle contact. Upon achieving a high-resistance seal, the electrode was capacitance-compensated. Membrane rupture was then achieved using the "zap" function while applying negative pressure, transitioning to whole-cell recording mode. After capacitance compensation, the recording mode was switched to voltage-clamp mode, with the clamp voltage set at − 90 mV. Pulses of depolarizing voltage steps from − 80 mV to + 120 mV in 10 mV increments, each lasting 500 ms, were applied to elicit outward currents corresponding to the membrane potential under test. The composition of the extracellular solution was as follows: 130 mmol·L^−1^ NaCl, 5 mmol·L^−1^ KCl, 1 mmol·L^−1^ MgCl2, 2 mmol·L^−1^ CaCl2, 10 mmol·L^−1^ HEPES, and 10 mmol·L^−1^ D-Glucose. The composition of the intracellular solution was as follows: 135 mmol·L^−1^ K-gluconate, 20 mmol·L^−1^ KCl, 2 mmol·L^−1^ EGTA, 3 mmol·L^−1^ Mg-ATP, 10 mmol·L^−1^ HEPES, 0.5 mmol·L^−1^ Na-GTP, and 5 mmol·L^−1^ QX-314 (Sigma-Aldrich, St. Louis, MO, USA). The pH is adjusted to 7.2 using KOH, and the osmolarity is adjusted to 300 mOsm. To inhibit calcium ion influx during cell adhesion, final concentration 10 μmol·L^−1^ of nifedipine (MKbio, Shanghai, China) was added both to the medium during cell seeding and to the extracellular solution. Data analysis was analyzed in IGOR.

Cells are seeded into 6-well plates at a density of 1 × 10^5^ cells per well for 24 h. Subsequently, Fluo-8 calcium fluorescent tracer was added to the complete culture medium to achieve a final concentration of 1 μmmol·L^−1^. The same concentration of nifedipine was added to inhibit calcium influx. After observation and recording of cellular fluorescence images using confocal microscopy, fluorescence quantification analysis was performed using ImageJ software.

### C-MYC cut&tag, MYC knocking down by shRNA and H3K27ac chip-seq

Auricular cartilage was obtained from patients undergoing tympanoplasty at the Eye and ENT Hospital of Fudan University. P1 and P3 cells were selected for cut&tag. The process of cut&tag high-throughput sequencing was performed as the standard protocol [80] (Novagene, Beijing, China) by c-MYC antibody (Proteintech, California, USA). After data quality control by software fastQC, the raw data was compared to GRCh38 using software BWA (V0.7.12). The peak calling and identifying genomic locations of regions were performed as previous in ATAC-seq.

After co-culturing P3 cells with 10 μmmol·L^−1^ nifedipine for 48 h, MYC knockdown was performed using a final concentration of 50 μmmol·L^−1^ shRNA (Hanbio, Shanghai, China).

Mouse H3K27ac chip-seq was downloaded from GEO database (GSE230235) and analyzed as previous described [81].

### Statistical analysis

All the data were presented as the means ± SD. Normality tests were conducted to confirm the normal distribution of data for parametric statistical analysis. Statistical differences were assessed using one way ANOVA or t-tests. All statistical analyses were performed with GraphPad Prism 10 (GraphPad Software, USA). A *p* value of less than 0.05 was considered statistically significant.

## Supplementary Information

Additional file 1: **Figure S1 A** Harmony integration of 9 samples. **B** Volcano plot of genes differentially expressed in 5 cell types. **C** Feature plot of marker genes in 5 cell types. **D** Volcano plot of differentially expressed genes between sub cell types ox-CDC and fc-CDC. **E** Heatmap and their GO terms foe CDC sub cell types. **F** Cell differentiation degree measured by CytoTrace. **G** Cell differentiation trajectories measured by Vector. **H** Heatmap and GO terms of genes differentially expressed in two branches predicted by monocle2. **I** Representative gene expression along with pseudo-time. (ox: oxidative; fc: functional; GO: Gene Ontology)

Additional file 2: **Figure S2 A** Heatmap of TF activity for 5 cell types. **B** RNA expression of top 15 TFs in each cell type. **C** Regulation network of representative TFs. **D** All TFs were clustered into 4 modules based on CSI. **D** Average TFs activity of each module were exhibited on feature plots (M1 and M3 mainly on SC/PHC, M2 mainly on CDC, M4 mainly on PROC/CPC. (SC: Stromal Cell, CDC: Chondrocyte Differentiated Cell, PHC: Pre-Hypertrophy Cell, PROC: Proliferative Cell, CPC: Chondrocyte Progenitor Cell; TF: Transcription Factor)

Additional file 3: **Figure S3 A** Volcano plot of differentially expressed genes among 3 cell passages. **B** Interaction network of genes enriched in oxidative phosphorylation (right). **C** EACR curves of 3 cell passages and 4 major parameters of ECAR.** D** Strength change of each cell-chat type from P1 to P3. **E** Number and interaction strength of cell-chat across cell passages.** F** Overall signaling patterns of cell-chat across cell passages. **G** Interaction strength change of FGF cell-chat across 3 cell passages. **H** Heatmap of FGF interaction strength with 5 cell types. **I** Expression of FGF subtypes for 3 cell passages. (ECAR: extracellular acidification rate * *p* < 0.05, ** *p* < 0.01, *** *p* < 0.001, **** *p* < 0.0001)

Additional file 4: **Figure S4 A-C** Heatmap, GO terms, KEGG pathways and representative gene expression difference in bulk transcriptome data1. **D-F** Bulk data2. **G-I** Bulk data3. **J** Umap of 3 scissor related cell groups. Scissor + means cell phenotypes correlated to P5 cells, Scissor- means cell phenotypes correlated to P0 cells. **K** Cell proportion of 5 cell types in scissor related subgroups. **L** Volcano plot of differentially expressed genes in 3 scissor related subgroups. Important marker genes were pointed in the plot.** M** GO terms of genes in 3 scissor subgroups. (Data1 was obtained from GEO database with GSE243387; Data2 and data3 were from article: Doi: 10.3390/cells8020085; *p < 0.05, **p < 0.01, ***p < 0.001, ****p < 0.0001, ns: no significance)

Additional file 5: **Figure S5 A** Collagen II staining for chondrogenic induction, alizarin red staining for osteogenic induction and oil red for adipogenic induction of P1, P3 and P6cells. **B** Crystal staining for monoclonal ability for P3 cells. (I: induced; N: non-induced)

Additional file 6: **Figure S6 A** Calcium channel protein expression in calcium signaling pathway. **B** ∆Current after adding nifedipine for P1 and P3 cells. **C** Vital genes expression along with measured pseudo-time. *D* Venn diagram of genes intersected between down-regulated in P3 + nifedipine and P1 cells of scRNA data. **E** Representative down-regulated genes and enriched TFs intersected by P1 cells and P3 + nifedipine. GO terms were marked. **F** RNA expression of SOX5, SOX6 and SOX9 after co-culture with nifedipine.** G** Alcian blue, safranin O and collagen II staining levels in cartilage pallets induced in vitro and in cartilage tissues induced in vivo. AverageOptical Density was measured by Image J. (GO: Gene Ontology; *p < 0.05, **p < 0.01, ***p < 0.001, ****p < 0.0001, ns: no significance)

Additional file 7: **Figure S7 A** MYC expression in human scRNA data. **B** *Myc* expression in mouse scRNA data. **C** MYC transcription network in SCENIC and relation between *MYC* and *SNED1/MGP*. **D** MYC single gene related set go terms and kegg pathways enrichment. **E** CALM1 and CALM2 expression in single-cell data. **F** Expression differences of CALM1/CALM2 between P3 and P3 + nifedipine cells. **G** Predicted mode of calmodulin binding to HuR. **H** Fluorescence staining confirms the colocalization of HuR and calmodulin. **I** RIP experiments indicated an increase in MYC binding with HuR after inhibiting calcium influx. **J** Distribution features of peaks. **K** MYC related motif. (RIP: RNA Immunoprecipitation; *p < 0.05, **p < 0.01, ***p < 0.001, ****p < 0.0001, ns: no significance)

Additional file 8.

## Data Availability

Single-cell RNA-seq data was uploaded to GEO database (GSE270642). ATAC-seq data was uploaded to GEO database (GSE270644). Bulk RNA-seq data was uploaded to GEO database (GSE270646). Cut&tag data was uploaded to GEO database (GSE270645). Bulk RNA-seq for stem cell was downloaded from GEO database (GSE57538). Mouse H3K27ac chip-seq was downloaded from GEO database (GSE230235).

## Abbreviations

SC: Stromal cell
CDC: Chondrocyte differentiated cell
PHC: Pre-hypertrophy cell
PROC: Proliferative cell
CPC: Chondrocyte progenitor cell
ox: Oxidative
fc: Functional
GO: Gene ontology
TF: Transcription factor
DEGs: Differentially expressed genes
WB: Western blot
OCR: Oxygen consumption rate
ECAR: Extracellular acidification rate
TSS: Transcription start sites
KEGG: Kyoto Encyclopedia of Genes and Genomes
HE: Hematoxylin and eosin
ECM: Extracellular matrix
