# Supplementary figures and images for: Blocking calcium-MYC regulatory axis inhibits early dedifferentiation of chondrocytes and contributes to cartilage regeneration
Source: Stem Cell Res Ther. 2025 Jul 15;16:372. doi: 10.1186/s13287-025-04483-3 (PMC12261834; doi:10.1186/s13287-025-04483-3)

A

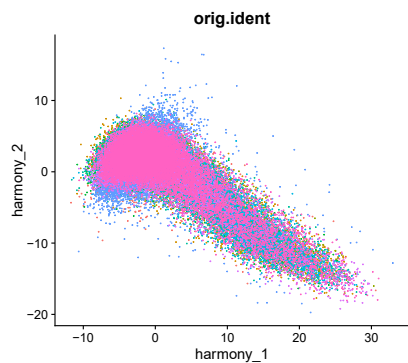

B

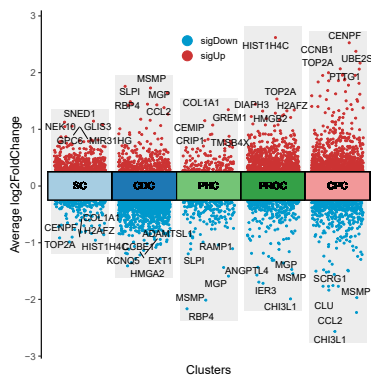

C

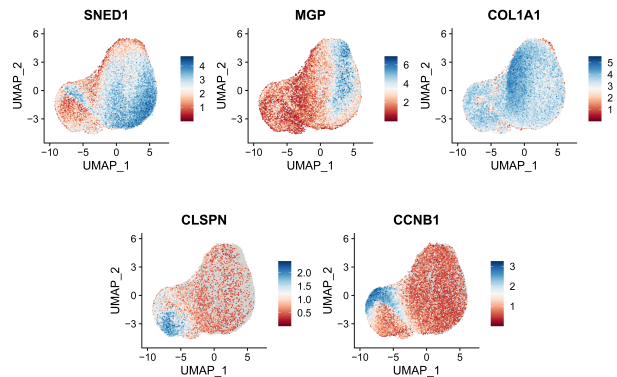

D

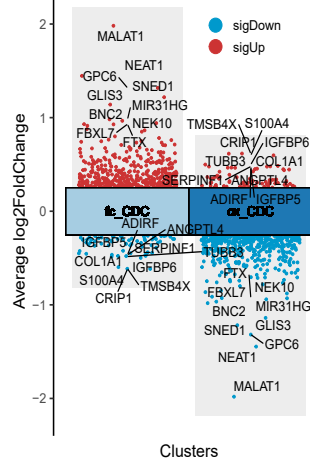

E

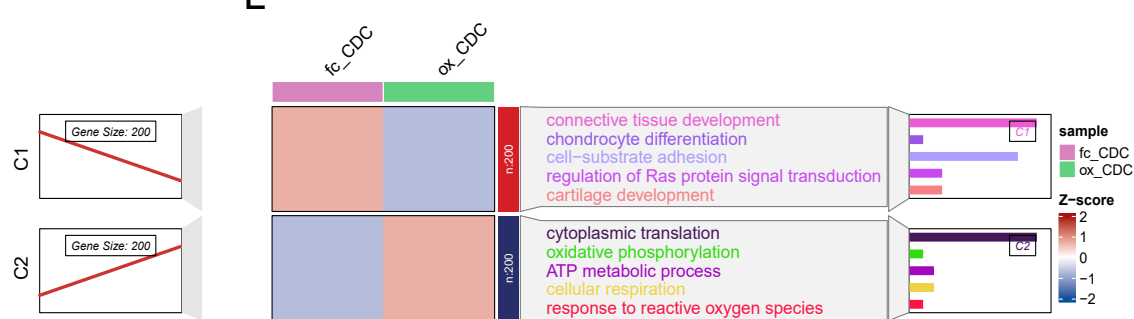

F

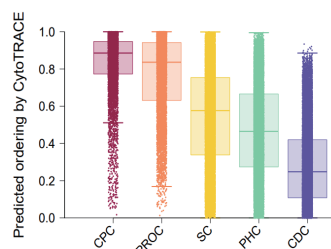

G

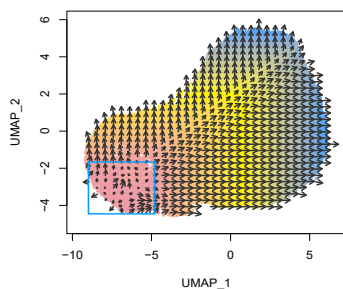

H

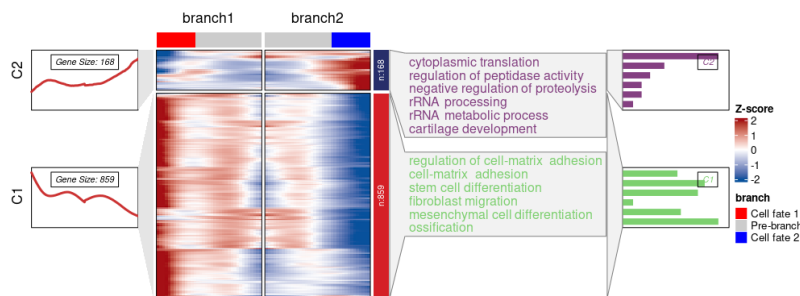

I

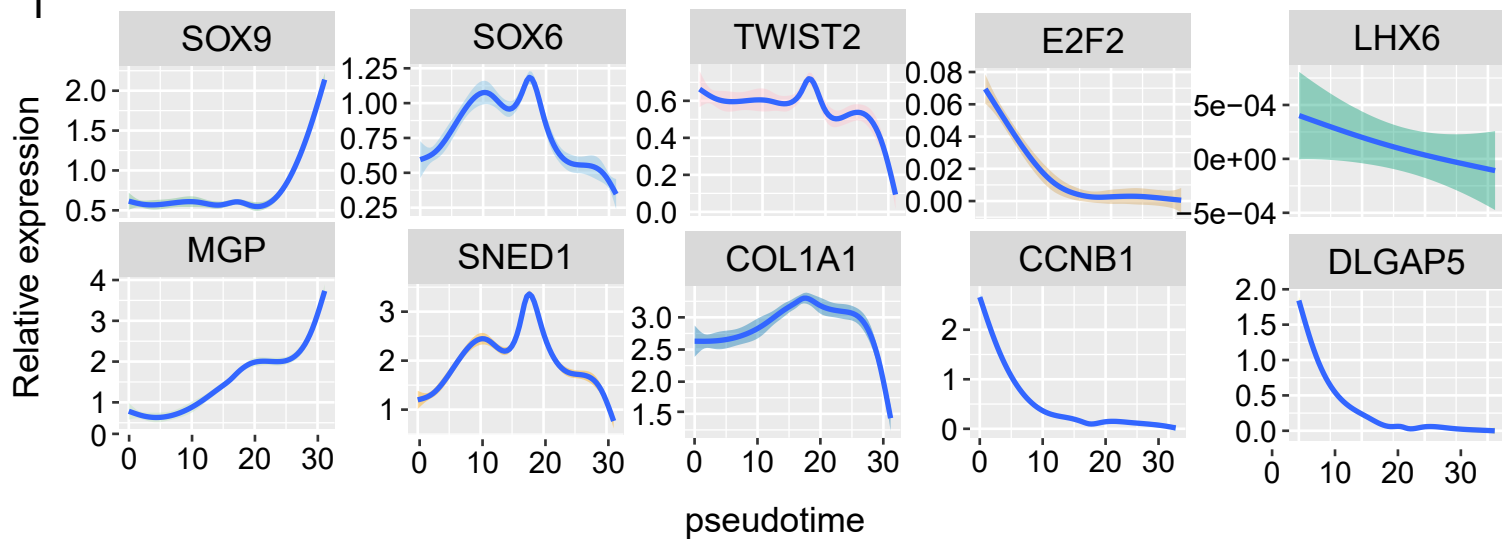

Supplement: Supplementary file 1 — Additional file 1: Figure S1 A Harmony integration of 9 samples. B Volcano plot of genes differentially expressed in 5 cell types. C Feature plot of marker genes in 5 cell types. D Volcano plot of differentially expressed genes between sub cell types ox-CDC and fc-CDC. E Heatmap and their GO terms foe CDC sub cell types. F Cell differentiation degree measured by CytoTrace. G Cell differentiation trajectories measured by Vector. H Heatmap and GO terms of genes differentially expressed in two branches predicted by monocle2. I Representative gene expression along with pseudo-time. (ox: oxidative; fc: functional; GO: Gene Ontology) [file 13287_2025_4483_MOESM1_ESM.pdf]

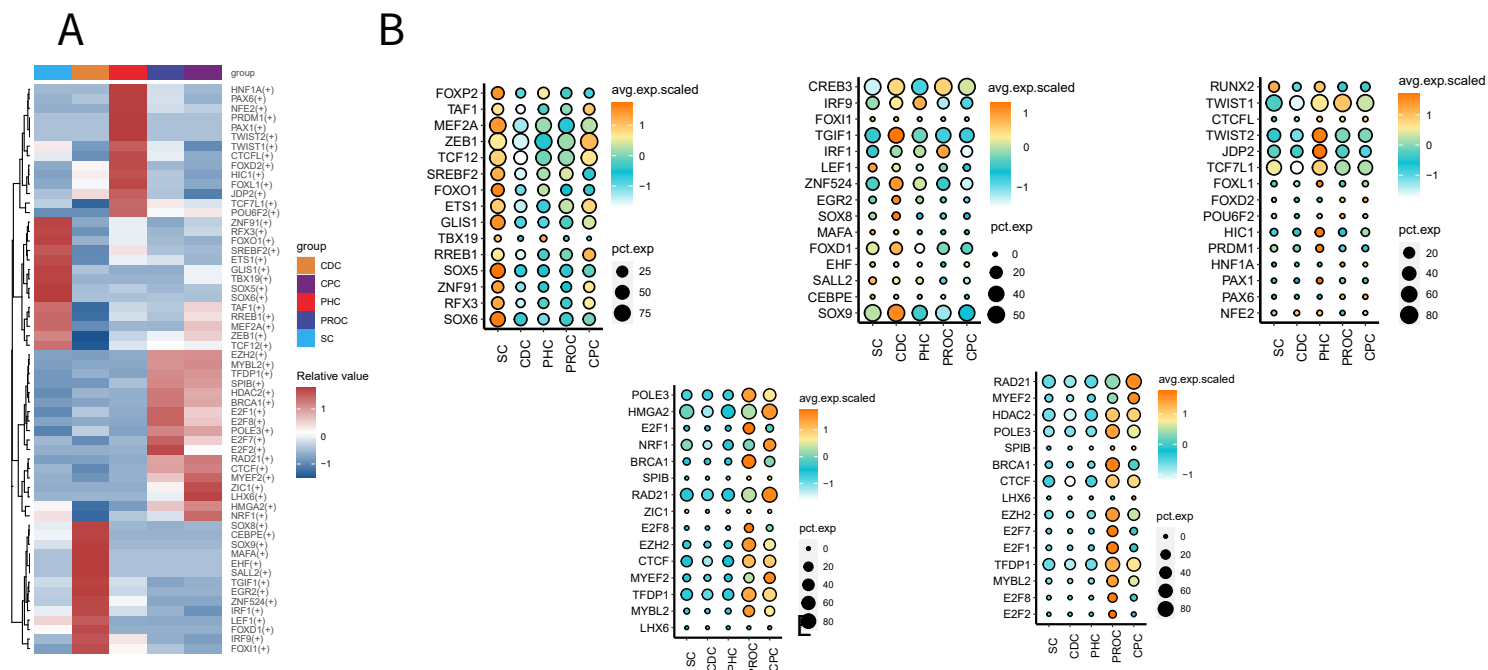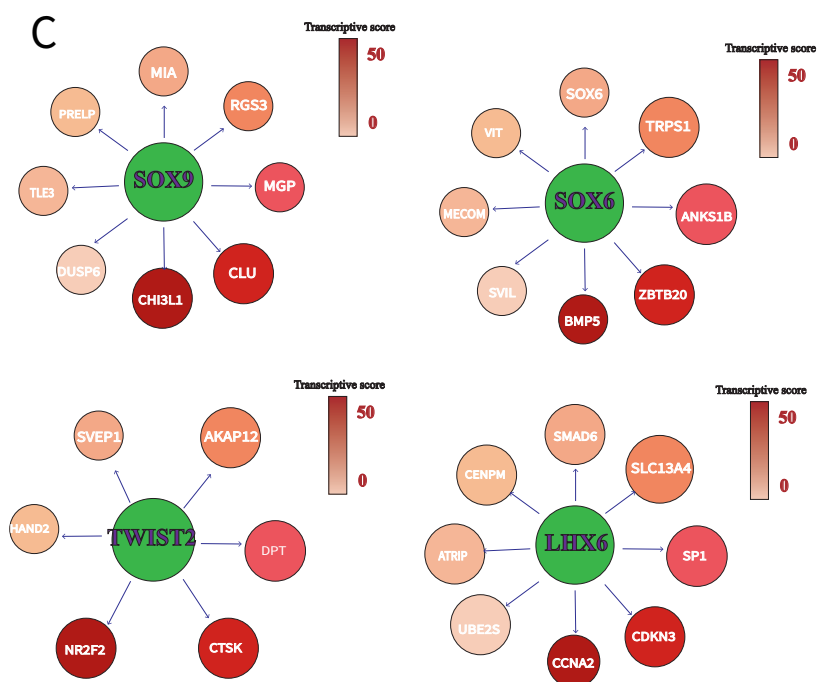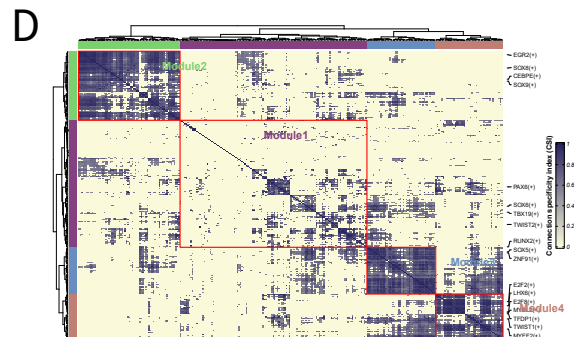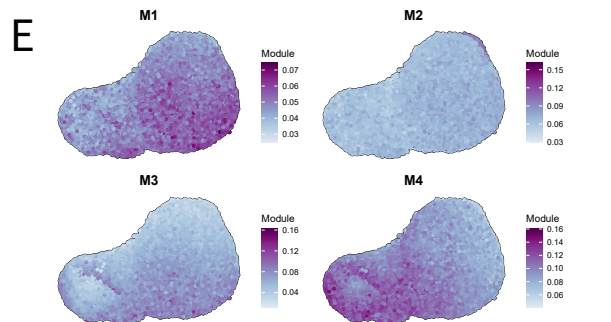

Supplement: Supplementary file 2 — Additional file 2: Figure S2 A Heatmap of TF activity for 5 cell types. B RNA expression of top 15 TFs in each cell type. C Regulation network of representative TFs. D All TFs were clustered into 4 modules based on CSI. D Average TFs activity of each module were exhibited on feature plots (M1 and M3 mainly on SC/PHC, M2 mainly on CDC, M4 mainly on PROC/CPC. (SC: Stromal Cell, CDC: Chondrocyte Differentiated Cell, PHC: Pre-Hypertrophy Cell, PROC: Proliferative Cell, CPC: Chondrocyte Progenitor Cell; TF: Transcription Factor) [file 13287_2025_4483_MOESM2_ESM.pdf]

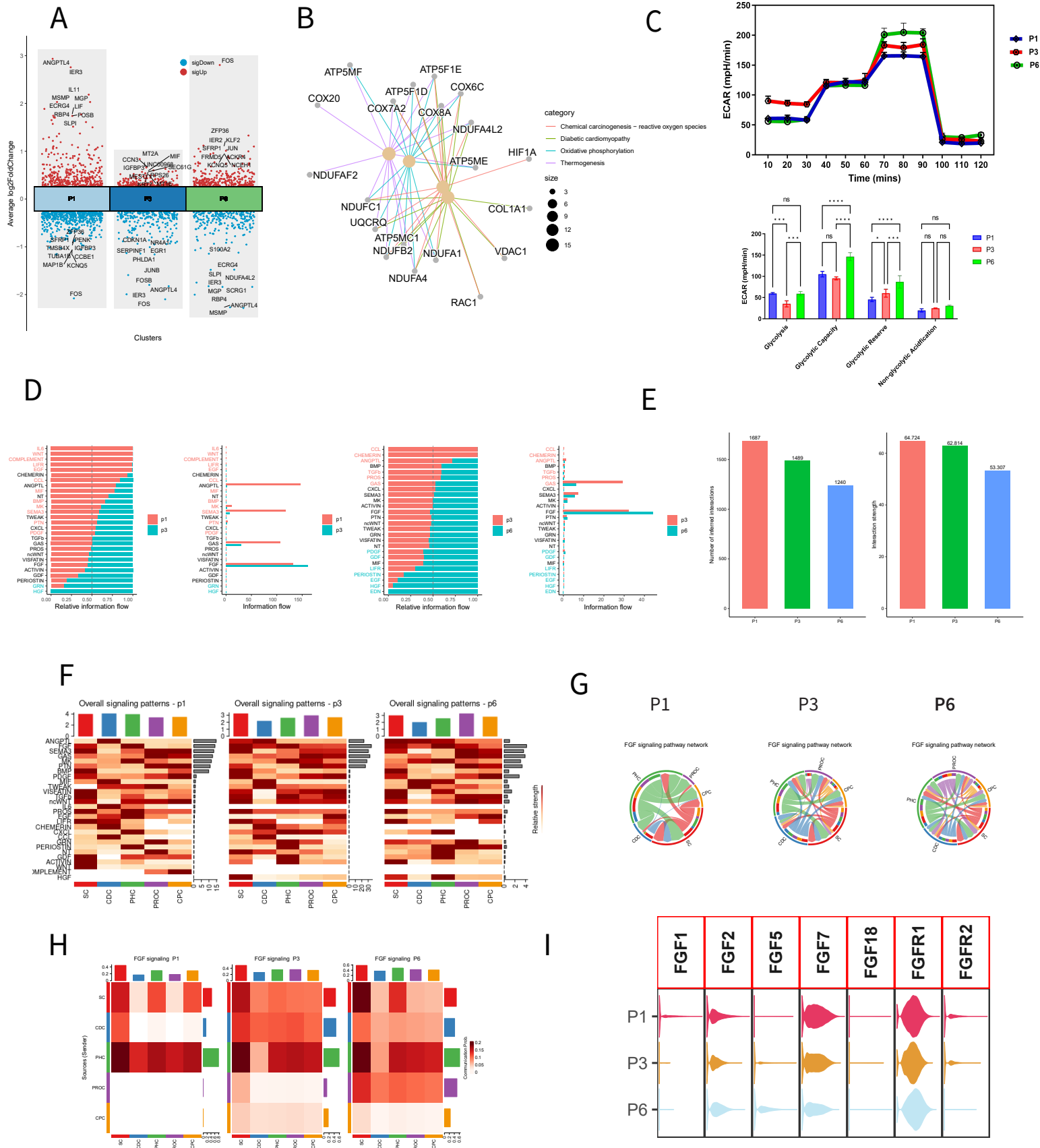

Supplement: Supplementary file 3 — Additional file 3: Figure S3 A Volcano plot of differentially expressed genes among 3 cell passages. B Interaction network of genes enriched in oxidative phosphorylation (right). C EACR curves of 3 cell passages and 4 major parameters of ECAR. D Strength change of each cell-chat type from P1 to P3. E Number and interaction strength of cell-chat across cell passages. F Overall signaling patterns of cell-chat across cell passages. G Interaction strength change of FGF cell-chat across 3 cell passages. H Heatmap of FGF interaction strength with 5 cell types. I Expression of FGF subtypes for 3 cell passages. (ECAR: extracellular acidification rate * p < 0.05, ** p < 0.01, *** p < 0.001, **** p < 0.0001) [file 13287_2025_4483_MOESM3_ESM.pdf]

## Bulk RNA transcriptome1

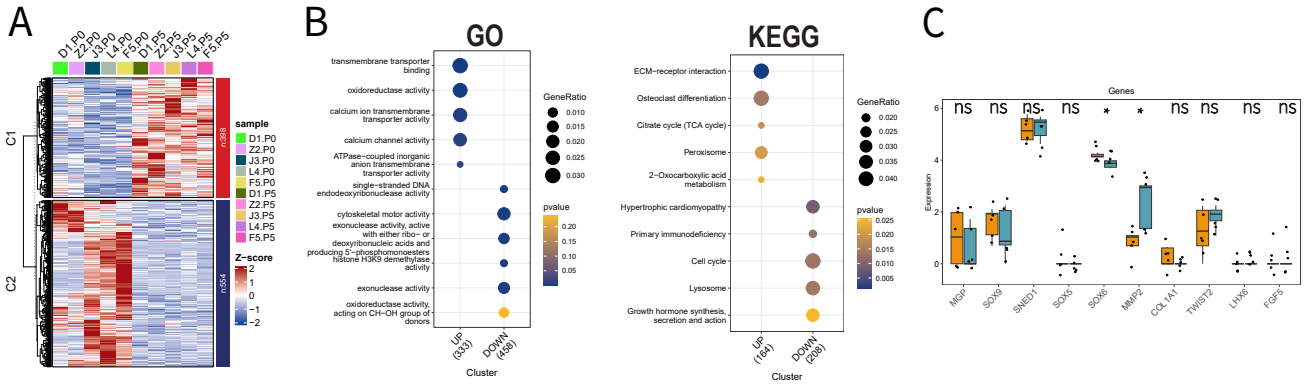

## Bulk RNA transcriptome2

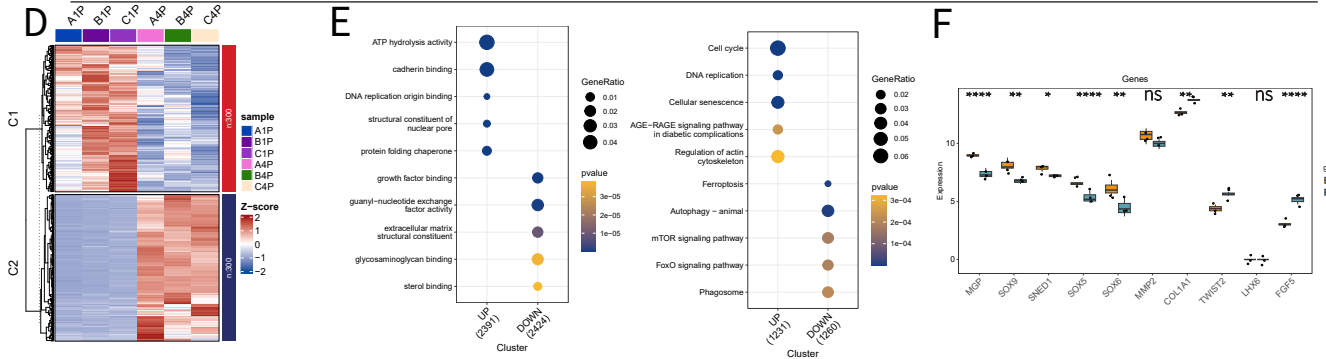

## Bulk RNA transcriptome3

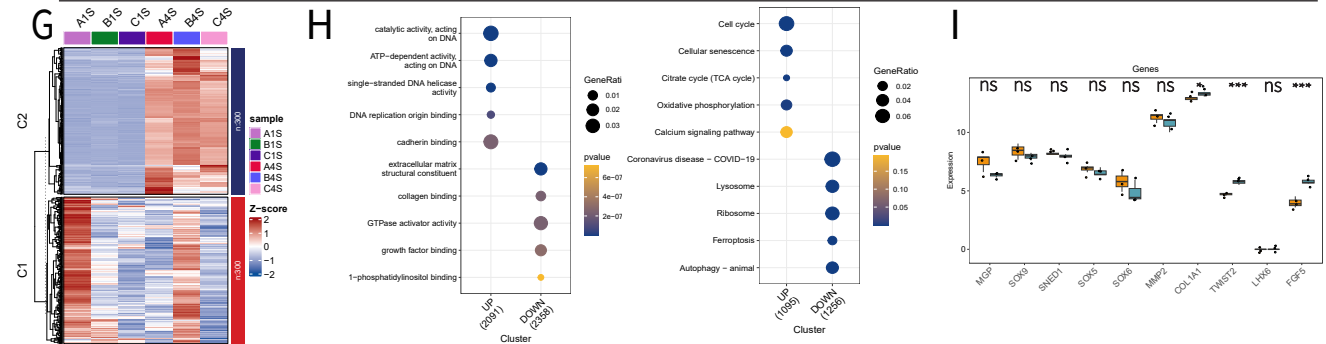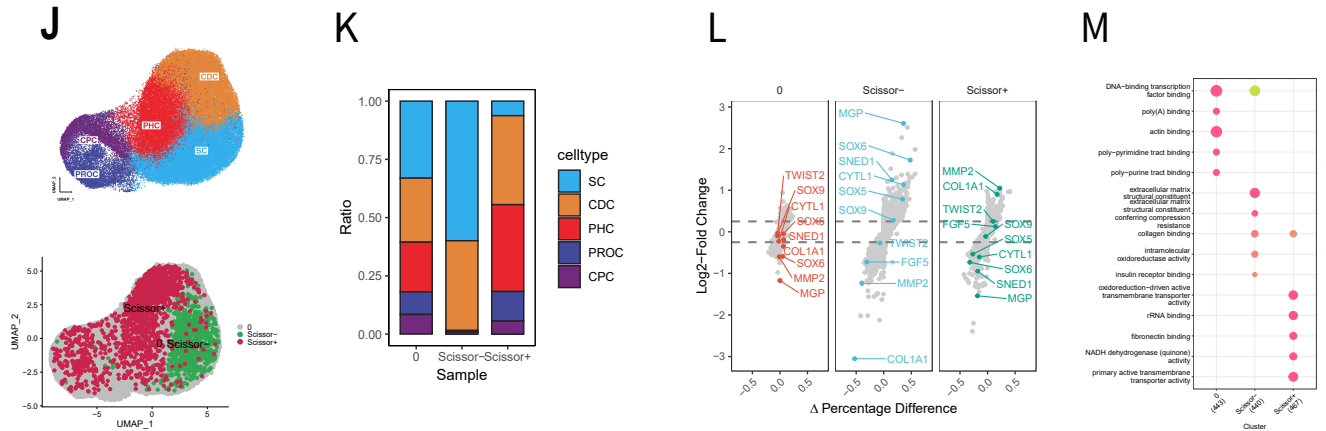

Supplement: Supplementary file 4 — Additional file 4: Figure S4 A-C Heatmap, GO terms, KEGG pathways and representative gene expression difference in bulk transcriptome data1. D-F Bulk data2. G-I Bulk data3. J Umap of 3 scissor related cell groups. Scissor + means cell phenotypes correlated to P5 cells, Scissor- means cell phenotypes correlated to P0 cells. K Cell proportion of 5 cell types in scissor related subgroups. L Volcano plot of differentially expressed genes in 3 scissor related subgroups. Important marker genes were pointed in the plot. M GO terms of genes in 3 scissor subgroups. (Data1 was obtained from GEO database with GSE243387; Data2 and data3 were from article: Doi: 10.3390/cells8020085; *p < 0.05, **p < 0.01, ***p < 0.001, ****p < 0.0001, ns: no significance) [file 13287_2025_4483_MOESM4_ESM.pdf]

A

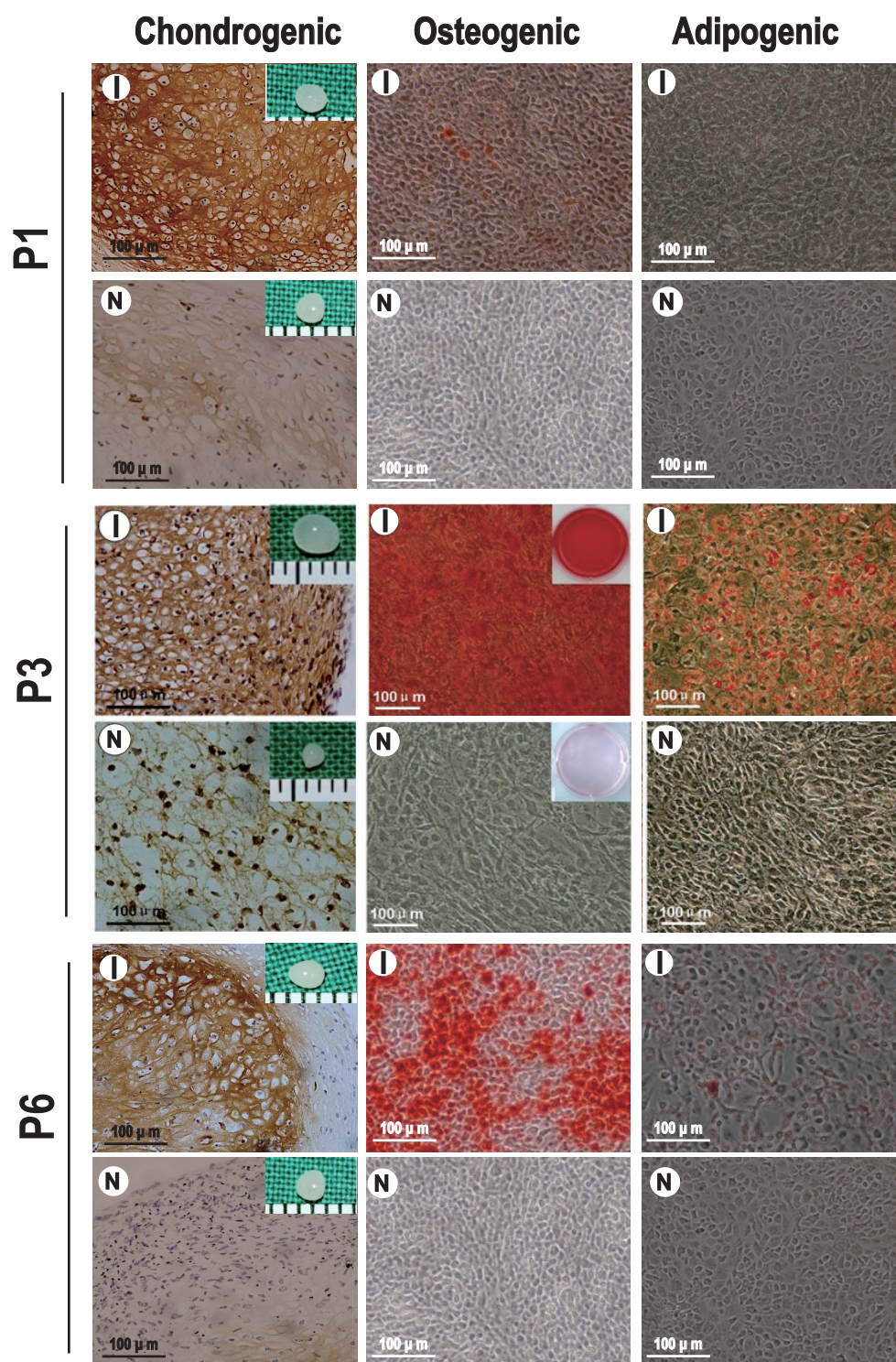

B

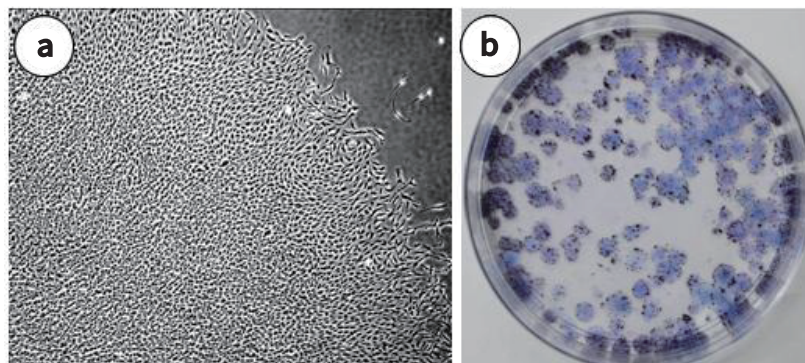

Supplement: Supplementary file 5 — Additional file 5: Figure S5 A Collagen II staining for chondrogenic induction, alizarin red staining for osteogenic induction and oil red for adipogenic induction of P1, P3 and P6cells. B Crystal staining for monoclonal ability for P3 cells. (I: induced; N: non-induced) [file 13287_2025_4483_MOESM5_ESM.pdf]

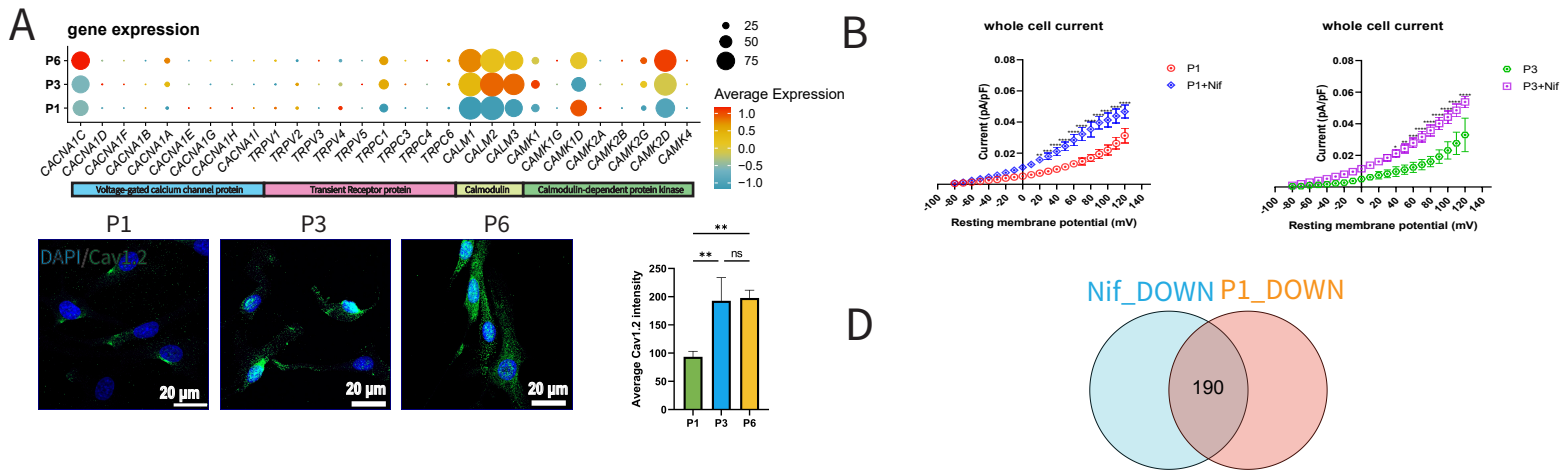

**G**

Cartilage pallets induced *in vitro*

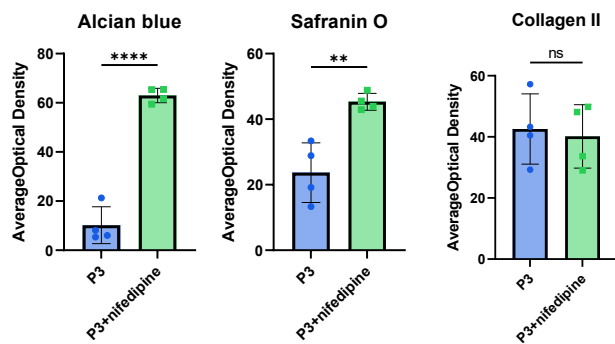

Cartilage tissues induced *in vivo*

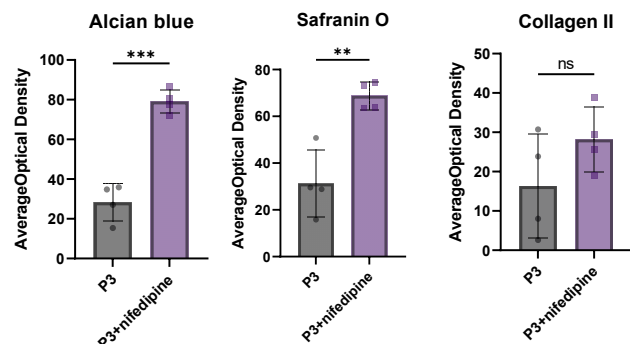

Supplement: Supplementary file 6 — Additional file 6: Figure S6 A Calcium channel protein expression in calcium signaling pathway. B ∆Current after adding nifedipine for P1 and P3 cells. C Vital genes expression along with measured pseudo-time. D Venn diagram of genes intersected between down-regulated in P3 + nifedipine and P1 cells of scRNA data. E Representative down-regulated genes and enriched TFs intersected by P1 cells and P3 + nifedipine. GO terms were marked. F RNA expression of SOX5, SOX6 and SOX9 after co-culture with nifedipine. G Alcian blue, safranin O and collagen II staining levels in cartilage pallets induced in vitro and in cartilage tissues induced in vivo. AverageOptical Density was measured by Image J. (GO: Gene Ontology; *p < 0.05, **p < 0.01, ***p < 0.001, ****p < 0.0001, ns: no significance) [file 13287_2025_4483_MOESM6_ESM.pdf]

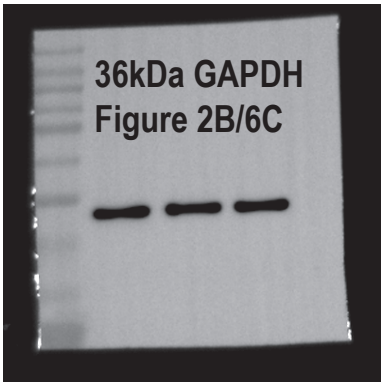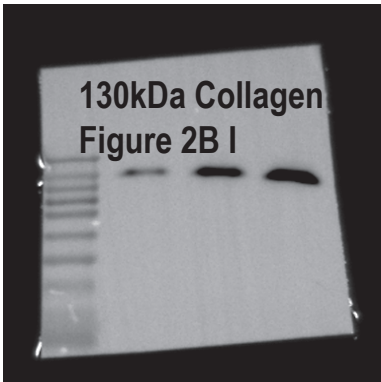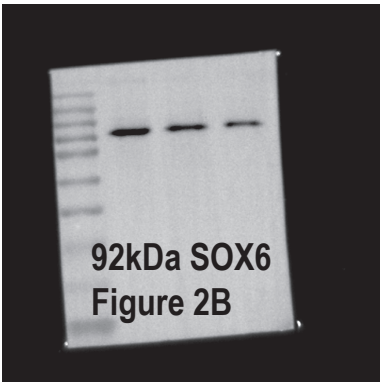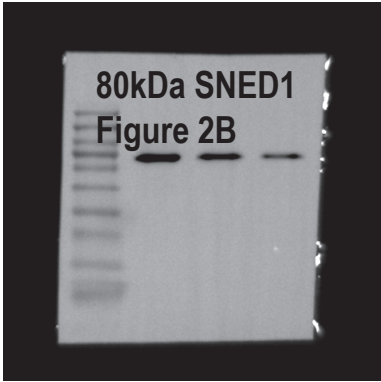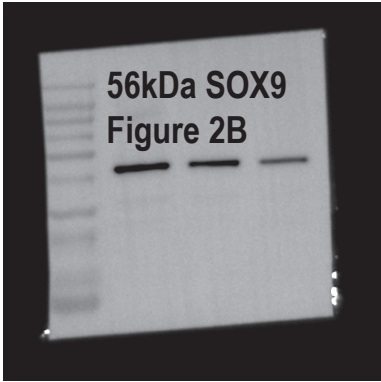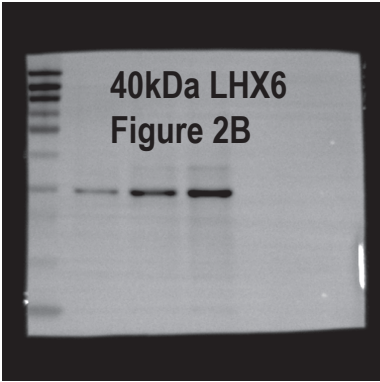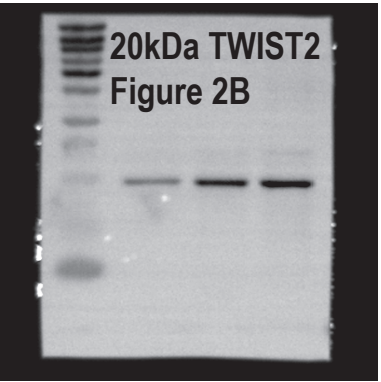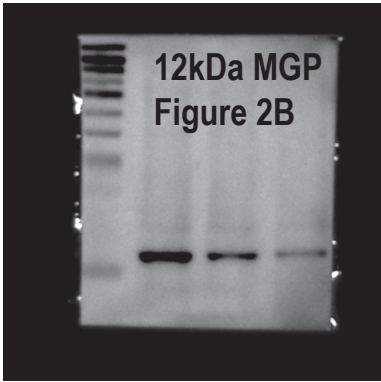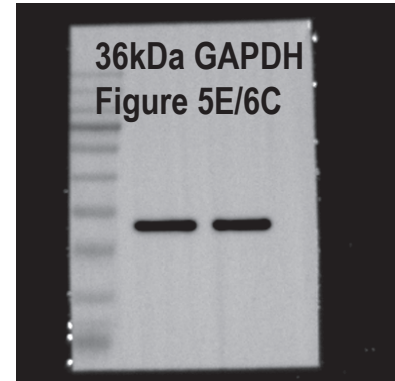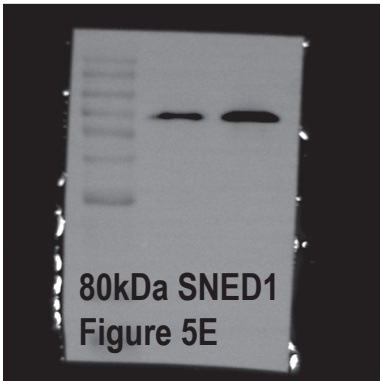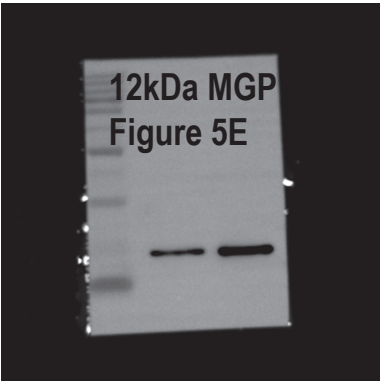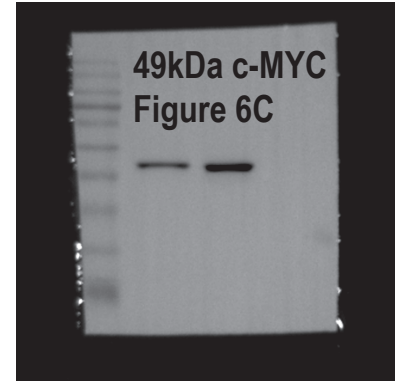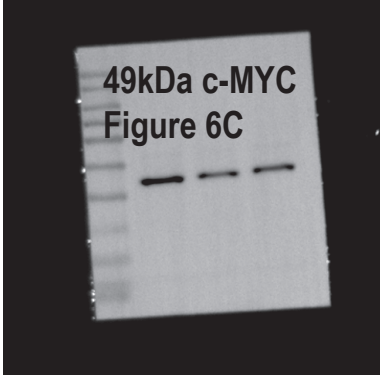

Supplement: Supplementary file 8 — Additional file 8. [file 13287_2025_4483_MOESM8_ESM.pdf]
