# Supplementary material for: Blocking calcium-MYC regulatory axis inhibits early dedifferentiation of chondrocytes and contributes to cartilage regeneration
Source: Stem Cell Res Ther. 2025 Jul 15;16:372. doi: 10.1186/s13287-025-04483-3 (PMC12261834; doi:10.1186/s13287-025-04483-3)
Supplement: Supplementary file 7 — Additional file 7: Figure S7 A MYC expression in human scRNA data. B Myc expression in mouse scRNA data. C MYC transcription network in SCENIC and relation between MYC and SNED1/MGP. D MYC single gene related set go terms and kegg pathways enrichment. E CALM1 and CALM2 expression in single-cell data. F Expression differences of CALM1/CALM2 between P3 and P3 + nifedipine cells. G Predicted mode of calmodulin binding to HuR. H Fluorescence staining confirms the colocalization of HuR and calmodulin. I RIP experiments indicated an increase in MYC binding with HuR after inhibiting calcium influx. J Distribution features of peaks. K MYC related motif. (RIP: RNA Immunoprecipitation; *p < 0.05, **p < 0.01, ***p < 0.001, ****p < 0.0001, ns: no significance) [file 13287_2025_4483_MOESM7_ESM.pdf]

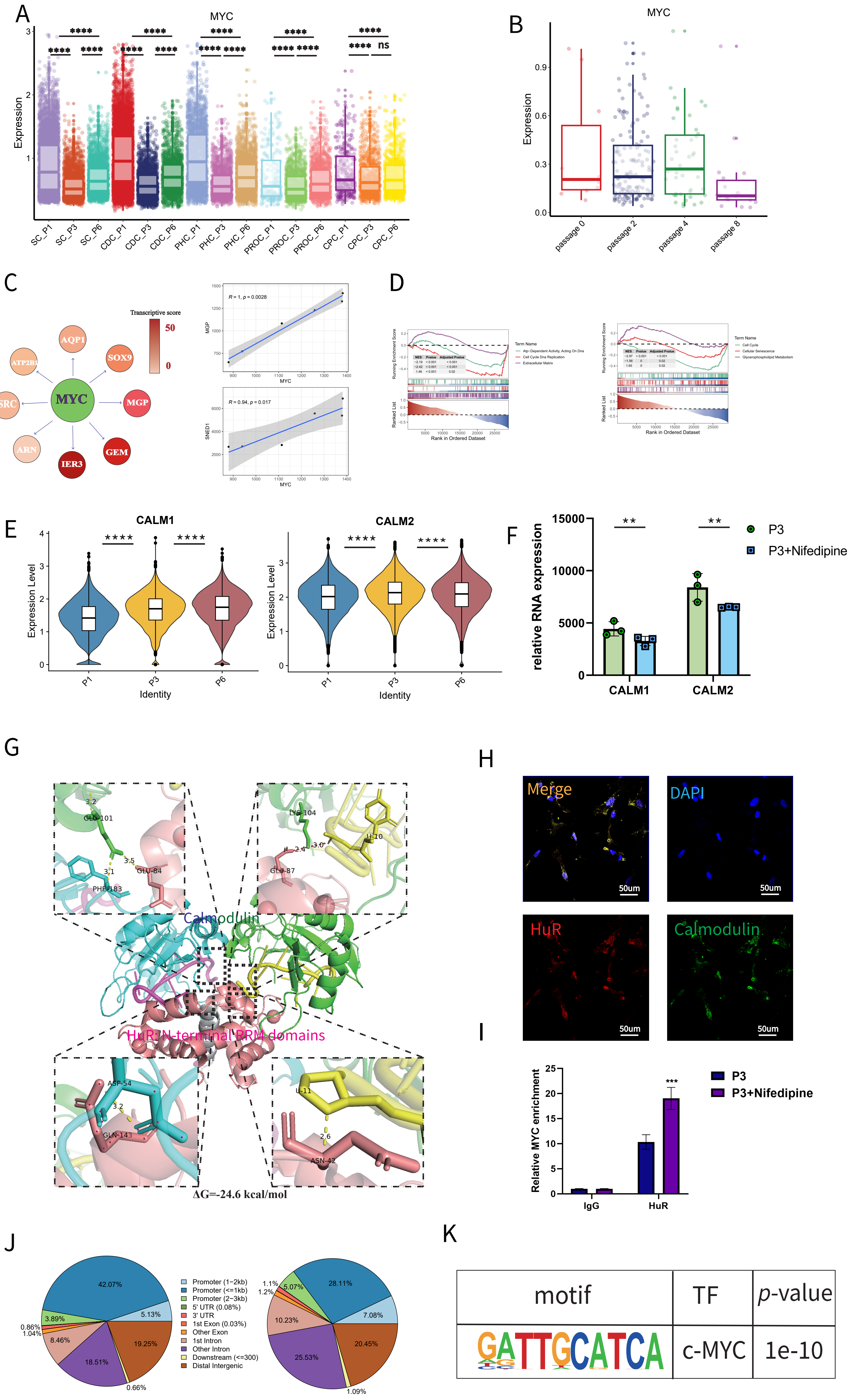
A
MYC
Expression
SC_P1
SC_P3
SC_P6
CDC_P1
CDC_P3
CDC_P6
PHC_P1
PHC_P3
PHC_P6
PROC_P1
PROC_P3
PROC_P6
CPC_P1
CPC_P3
CPC_P6
ns
B
MYC
Expression
passage 0
passage 2
passage 4
passage 8
C
Transcriptive score
AQP1
SOX9
ATP2B1
MYC
SRC
MGP
ARN
GEM
IER3
R = 1, p = 0.0028
R = 0.94, p = 0.017
MGP
SNED1
MYC
D
Running Enrichment Score
Ranked List
Rank in Ordered Dataset
Term Name
Atp-Dependent Activity, Acting On Dna
Cell Cycle Dna Replication
Extracellular Matrix
Cell Cycle
Cellular Senescence
Glycerophospholipid Metabolism
E
CALM1
CALM2
Expression Level
Identity
P1
P3
P6
F
relative RNA expression
P3
P3+Nifedipine
CALM1
CALM2
G
GLU-101
GLU-84
PHE-183
LYS-104
U-10
GLU-87
Calmodulin
HuR: N-terminal RRM domains
ASP-54
GLN-143
U-11
ASN-42
ΔG=-24.6 kcal/mol
H
Merge
DAPI
HuR
Calmodulin
50um
I
Relative MYC enrichment
P3
P3+Nifedipine
IgG
HuR
J
Promoter (1~2kb)
Promoter (<=1kb)
Promoter (2~3kb)
5' UTR (0.08%)
3' UTR
1st Exon (0.03%)
Other Exon
1st Intron
Other Intron
Downstream (<=300)
Distal Intergenic
42.07%
5.13%
19.25%
18.51%
8.46%
3.89%
0.86%
1.04%
0.66%
28.11%
7.08%
20.45%
25.53%
10.23%
5.07%
1.1%
1.2%
1.09%
K
motif
TF
p-value
GATTGCATCA
c-MYC
1e-10
